# Chemical Modification of 2,6,9-Trisubstituted Purine CDK Inhibitors: *tert*-Butylation at *N*7/*N*9 and Access to 2,6,7-Trisubstituted Analogs

**DOI:** 10.1021/acsomega.5c10284

**Published:** 2026-04-14

**Authors:** Michal Valenta, Jakub Stýskala

**Affiliations:** Department of Organic Chemistry, Faculty of Science, Palacký University, 17. listopadu 12, 771 46 Olomouc, Czech Republic

## Abstract

This study focuses on the chemical modification of known 2,6,9-trisubstituted purine cyclin-dependent kinase (CDK) inhibitors as model compounds, with particular emphasis on the incorporation of the less-explored *tert*-butyl group (representing *tert*-alkyl substituents) at the *N*7 and *N*9 positions of the purine core to prepare 2,6,7- and 2,6,9-trisubstituted analogs. Primarily chemical aspects of these modifications are studied, focusing on the effect of the *tert*-butyl group on chlorine reactivity at *C*2/*C*6 position and purine ring stability during substitution reactions, especially in the case of 2,6,7-trisubstituted compounds. The effect of the *tert*-butyl group is also evaluated in comparison with other substituents at the purine ring. Furthermore, a novel and unambiguous synthetic route to key intermediates −7 and 9-(*tert*-butyl)-2,6-dichloropurines-has been developed by a new method to enable targeted substitution at the chlorine-bearing positions.

## Graphical abstract

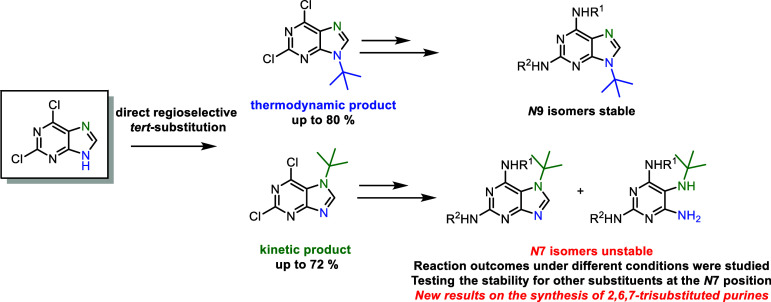

## Introduction

2,6,9-Trisubstituted purines represent a well-established class of bioactive compounds with broad pharmacological relevance, particularly in antiviral, anticancer, and anti-inflammatory therapies. Their substitution pattern allows precise modulation of biological activity and target specificity. Among these compounds are purine-based CDK inhibitors,^1–3^ which play a critical role as key regulators of the eukaryotic cell cycle—notable examples include olomoucine, roscovitine (Seliciclib), bohemine, and purvalanol A.^4–12^

In contrast, 2,6,7-trisubstituted purines have received significantly less attention, yet they offer unique structural and functional potential. The introduction of a substituent at position *N*7 can lead to altered conformational properties and novel biological interactions.^13^

In this work we aimed to modify the mentioned compounds by replacing the original substituents at the *N*7 and *N*9 positions of the purine ring with a less-explored *tert*-butyl group, which remains underrepresented in medicinal purine chemistry (Figure 1). This limitation is primarily attributed to the difficulty of preparing suitable purine precursors, as classical substitution methods are generally ineffective for the introducing tertiary groups.^14^ We were interested in whether, and how, the position of the *tert*-butyl group influences mainly chemical and a lesser extent biological effects.

**Figure 1:**
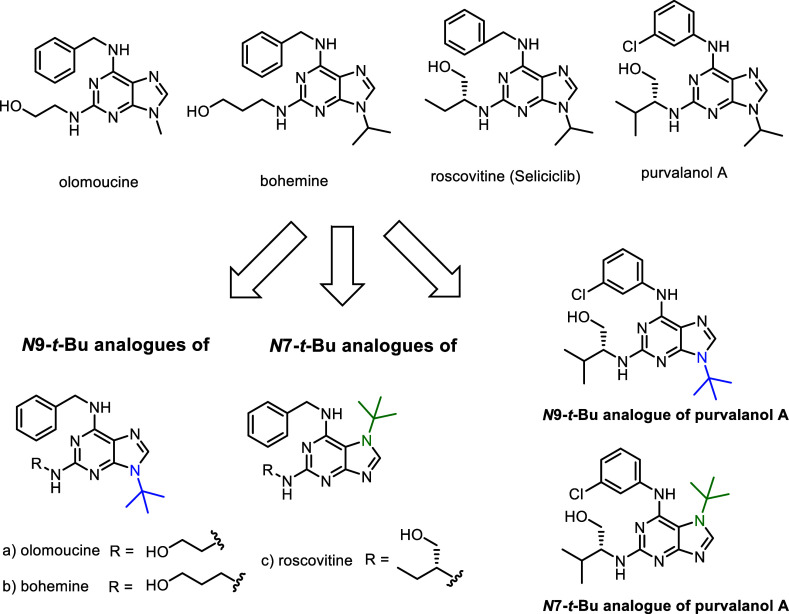
Targeted *N*7/*N*9 *tert*-butylated analogues of known CDK inhibitors.

## Results and Discussion

The key intermediates for the intended chemical and biological studies were 7- and 9-(*tert*-butyl)-2,6-dichloropurines **2** and **3**. These isomeric precursors are known and were previously described as products of the reaction of 2,6-dichloropurine with *tert*-butanol over alumina, affording a mixture that required chromatographic separation to isolate the pure compounds. Unfortunately, the reported yields were low (15% for the *N*7 isomer and 24% for the *N*9 isomer).^15^ Based on our recently published results enabling the direct introduction of a *tert*-alkyl group at the *N*7 position of the purine ring,^14^ we adapted these conditions for the reaction with 2,6-dichloropurine and identified a significant improvement and straightforward route to both the *N*7 isomer **2** and the *N*9 isomer **3**, obtained in high yield. This method, derived from Vorbrüggen’s glycosylation of purines,^16–21^ is based on the reaction of *N*-trimethylsilylated purines with a *tert*-alkyl halide in the presence of SnCl_4_ as a catalyst. Depending on the reaction conditions, both kinetic and thermodynamic product can be obtained.

The *tert*-butylation of 2,6-dichloropurine was performed under previously established conditions, using 1 equiv of purine, 2.1 equiv of SnCl_4_ as catalyst, and 3 equiv of *tert*-butyl bromide (*t*-BuBr) in either DCE (1,2-dichloroethane) or acetonitrile (ACN). In general, we found that the more polar solvent ACN provided better conversion to the desired products compared to DCE, where the reaction proceeded more slowly. Therefore, further optimization reactions were carried out in ACN (Scheme 1).

**Scheme 1:**
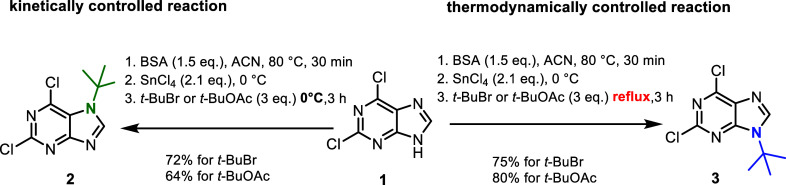
Preparation of the *N*7/*N*9 *tert*-Butylated 2,6-Dichloropurines **2** and **3**

Temperature and reaction time were found to be critical factors in the selective preparation of the kinetic *N*7 isomer **2** and the thermodynamic *N*9 isomer **3**. To minimize the formation of side products, the optimal conditions for obtaining the kinetic *N*7 isomer **2** involved a reaction temperature of 0 °C and a reaction time of 3 h. Prolonged reaction times and elevated temperatures (even room temperature) led to the formation of undesired isomers. Under these optimized conditions, simple aqueous workup afforded a crude product **2** in 72% yield and sufficient purity. Notably, the originally reported washing step with aqueous NaHCO_3_ had to be omitted, as it led to the formation of additional byproducts.

The thermodynamically more stable *N*9 isomer **3** was obtained by heating the reaction mixture under reflux for 3 h, affording a 75% yield. These conditions were sufficient to achieve complete conversion of the less stable isomers formed during the *tert*-butylation reaction.

Although the degree of conversion was not always quantitative under all conditions, the unreacted starting material **1** could be easily removed by aqueous extraction, yielding crude products with very good purity. This method is suitable for the large-scale synthesis of both isomers, making it a practical approach for further development or application.

We found that the regioselective *tert*-butylation of 2,6-dichloropurine can also be performed under the described conditions using *tert*-butyl acetate instead of the toxic *tert*-butyl bromide, affording comparable results.

The position of the *tert*-butyl group in the prepared isomers was confirmed by ^13^C NMR spectroscopy (Figure 2). For the *N*7 isomer **2**, a characteristic chemical shift of the *C*5 atom in the purine ring was observed at approximately 122 ppm, which is in agreement with values reported for *N*7 substituted 6-chloropurines.^22–24^ In contrast, the corresponding signal for the analogous *N*9 substituted 6-chloropurines appeared at around 132 ppm.^25,26^

**Figure 2:**
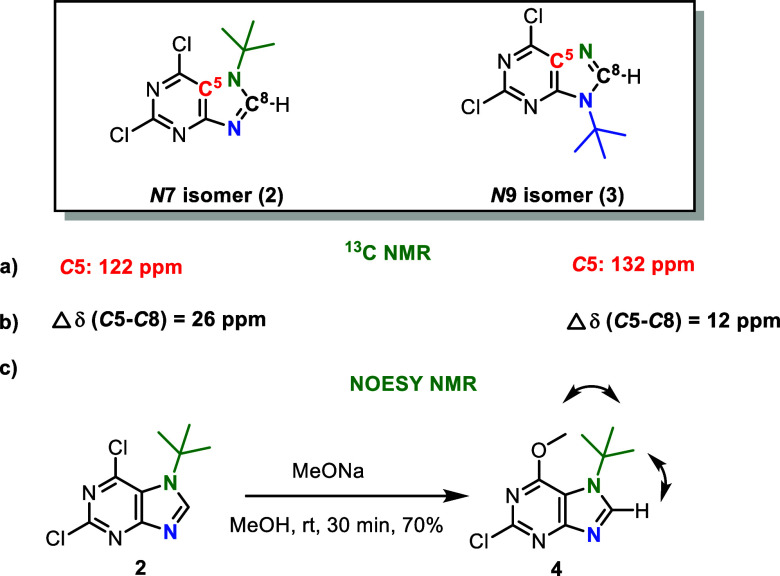
Resolution of *N*7/*N*9 regioisomers **2** and **3** based on (a) the chemical shift of the *C*5 carbon atom, (b) difference between the chemical shifts of the *C*5 and *C*8 carbon atoms, and (c) NOESY NMR experiment.

The difference between the chemical shifts of the *C*5 and *C*8 atoms (Δδ) was also consistent with previously published data, which are commonly used to distinguish between purine *N*7/*N*9 isomers: for the *N*7 isomer **2** higher, Δδ = 26; for the *N*9 isomer **3** smaller, Δδ = 12.^27,28^

Further confirmation of the *N*7 substitution in compound **2** was obtained via a NOESY NMR experiment after derivatization at the *C*6 position with a methoxy group, yielding compound **4**, where characteristic NOE interactions were observed (Figure 2). Additionally, the ^1^H NMR data matched previously published values for compounds **2** and **3**, which were prepared using alumina as a catalyst, where only ^1^H NMR spectra were reported.^15^

The prepared *N*7/*N*9 *tert*-butylated 2,6-dichloropurines **2** and **3** were used to modify the structure of the above-mentioned CDK inhibitors at the imidazole ring. The synthesis of these derivatives was based on classical nucleophilic substitution of the chlorine atoms in the corresponding *tert*-butylated purine precursors.

In the case of the *N*9-*t*-Bu isomers, no issues were encountered, and the desired compounds **7a**–**c** and **8** were obtained in good yields (Scheme 2). The starting *N*9 precursors **5** and **6** were synthesized by nucleophilic substitution of the more reactive chlorine atom at position 6 of 9-(*tert*-butyl)-2,6-dichloropurine (**3**) with benzylamine and 3-chloroaniline, respectively. No significant difficulties were observed even with the less nucleophilic 3-chloroaniline. The resulting intermediates were further modified via substitution of the chlorine atom at position 2 with the corresponding amino alcohols to afford the target 9-*tert*-butylated 2,6-disubstituted purines **7a**–**c** and **8**. These reactions were carried out at 150 °C using 8 equiv of the respective amino alcohol, which also served as the solvent.

**Scheme 2:**
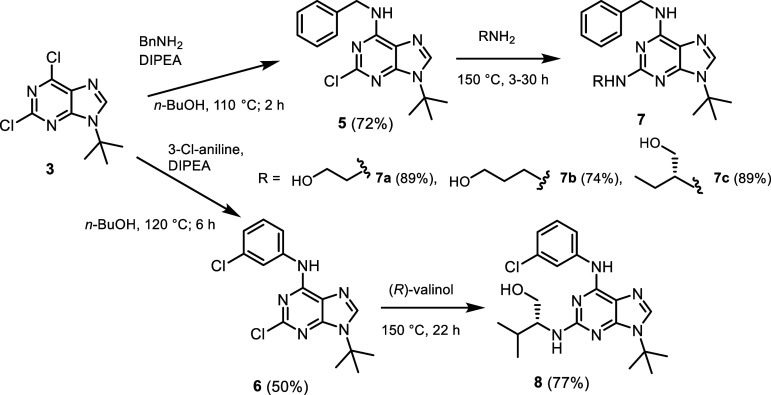
Preparation of *N*9 *tert*-Butylated Analogues of CDK Inhibitors **7a**–**c** and **8**

In contrast, the synthesis of the *N*7-*t*-Bu analogues **11a**–**c** and **13** proved challenging, even after extensive optimization. Several unexpected side reactions associated with the *tert*-butyl group at the *N*7 position of the purine ring were observed.

For the preparation of *N*7 precursors **9** and **10**, we found that classical substitution with the less nucleophilic 3-chloroaniline failed, in contrast to the successful reaction with benzylamine. Neither elevated temperature, prolonged reaction time, microwave irradiation, the use of a specialized InCl_3_ catalyst,^29^ nor Buchwald–Hartwig amination led to product formation (Scheme 3). This observation is consistent with a previously reported unsuccessful attempt to react 4-methoxyaniline with 7-(*tert*-butyl)-6-chloropurine, where the problem remained unresolved.^14^ To overcome this limitation, we attempted to enhance the nucleophilicity of 3-chloroaniline by converting it into the corresponding formamide and carrying out the substitution in the presence of NaH. This approach afforded the *N*7 precursor **10** in 38% yield. Notably, a structurally related *N*7 methyl derivative employing 3-chloroaniline was previously synthesized under classical mild conditions.^30^

**Scheme 3:**
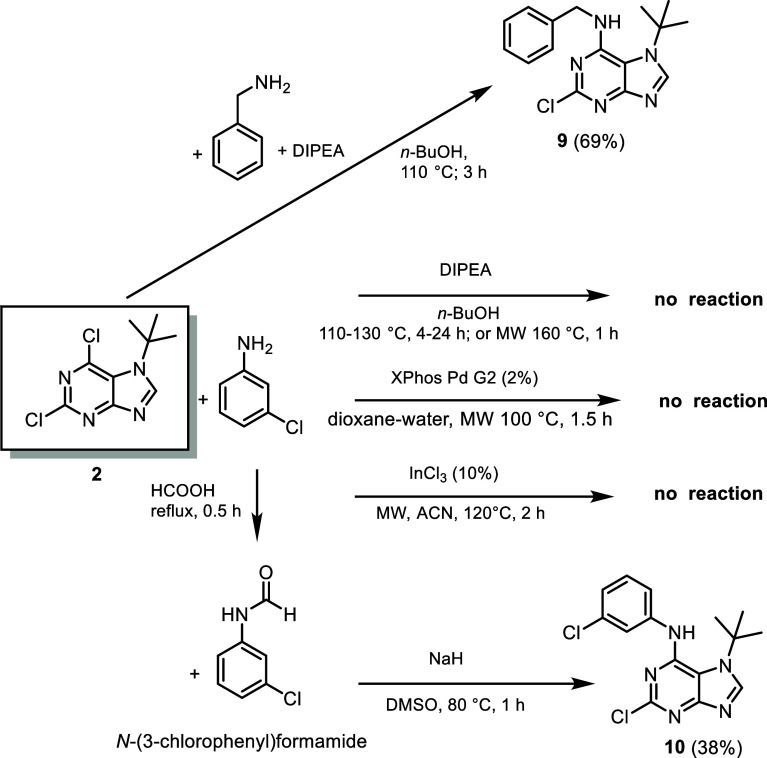
Preparation of *N*7 *tert*-Butylated Precursors **9** and **10**

During the synthesis of *N*7-*t*-Bu analogues, an additional unexpected reaction was observed upon substitution of the chlorine atom at position 2 with amino alcohols. Under similar reaction conditions (150 °C, solvent-free), the reaction yielded a mixture of products, with the major component exhibiting a molecular ion 10 *m*/*z* units lower than the expected compounds **11** or **13**, respectively (Scheme 4). With increasing reaction time, complete conversion of the starting compound **9** was observed, followed by a gradual transformation of the desired products **11** into undesired byproducts **12** (Table 1, entries 1 and 2). Isolation and structural analysis of these byproducts revealed that they arise from ring-opening of the imidazole moiety, accompanied by the loss of a carbon atom. Experimental data clearly indicate that the formation of these side products is both temperature- and time-dependent. It is also evident that the desired products are initially formed and subsequently degrade, leading to the formation of the ring-opened imidazole derivatives. A proposed reaction mechanism is shown in Scheme 5.

**Scheme 4:**
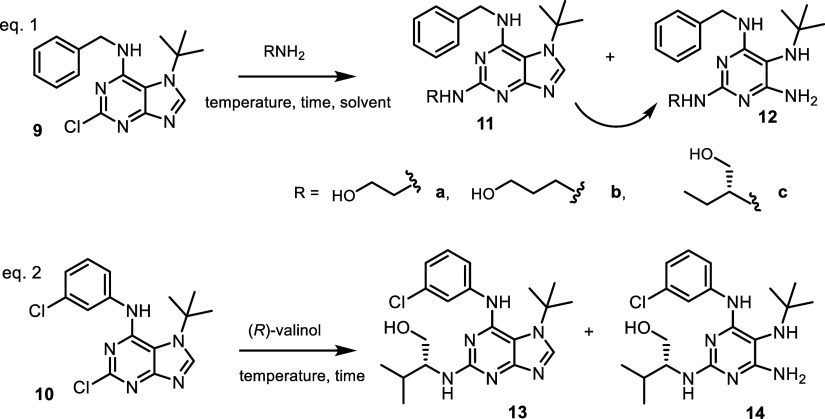
Preparation of *N*7 *tert*-Butylated Analogues of CDK Inhibitors **11a**–**c** and **13**

**Table 1:** Optimalization Reactions Leading to *N*7-*t*-Bu Analogues of CDK Inhibitors **11a**–**c** and **13**

entry	reaction with	aminoalcohol (equiv)	temperature (°C)	time (h)	solvent	ratio of 9:11a:12a %^a^
1	2-aminoethanol and **9**	8	150	2		10:25:65
2		8	150	6		0:20:80
3		8	100	29		10:30:60^b^
4		**20**	**100**	**11**		**10:80:10** ^c^
5		20	100	4	*n*-BuOH	95:5:0
6		20	100	20	*n*-BuOH	70:30:0
7		20	110	20	*n*-BuOH	38:57:5
8		20	150	15	*n*-BuOH	0:40:60
9		20	100	20	anisole	94:6:0
10		20	110	20	anisole	86:13:1
11		20	150	15	anisole	60:15:25
12		20	100	20	DMSO	72:28:0
13		20	110	20	DMSO	39:59:2
14		20	150	15	DMSO	0:60:40
						ratio of **9:11** **b:12b** %
15	3-aminopropanol and **9**	8	100	29		5:45:50^d^
16		**20**	**100**	**14**		**4:80:16** ^e^
						ratio of **9:11** **c:12c** %
17	(*R*)-3-aminobutanol and **9**	8	130	29		0:24:76
18		**20**	**130**	**6**		**38:45:17** ^f^
						ratio of **10:13:14**%
19	(*R*)-valinol and **10**	8	150	2		5:30:65
20		20	160	6		0:5:95^g^
21		20	100	3		94:5:1
22		**20**	**120**	**6**		**30:37:33** ^h^

a Based on LC/MS analysis.

b The isolated yield of **12a** was 59% (by semiprep HPLC).

c The isolated yield of **11a** was 54% (by semiprep HPLC).

d The isolated yield of **12b** was 52% (by semiprep HPLC).

e The isolated yield of **11b** was 48% (by semiprep HPLC).

f The isolated yield of **11c** was 31% (by semiprep HPLC).

g The isolated yield of **14** was 43% (by semiprep HPLC).

h The isolated yield of **13** was 24% (by semiprep HPLC).

**Scheme 5:**
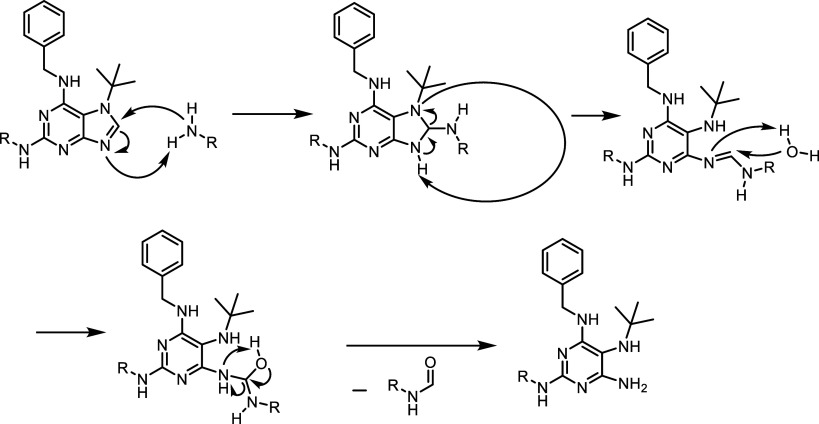
Proposed Mechanism for the Formation of the Ring-Opened Imidazole Derivative

To suppress the competing side reaction, a series of optimization experiments were conducted by varying the reaction temperature, the amount of amino alcohol, and the choice of solvent. In general, performing the reaction at a lower temperature (100 °C) partially alleviated the formation of the undesired byproduct. However, this introduced a new complication–significantly prolonged reaction times at lower temperatures negatively impacted the overall outcome, as they ultimately favored the formation of the side product (Table 1, entry 3).

Improved results were obtained by increasing the amount of amino alcohol from 8 to 20 equiv at 100 °C, which effectively suppressed the side reaction to an acceptable extent (Table 1, compare entries 3 and 4 or 15 and 16). Nevertheless, for the synthesis of *N*7-*t*-Bu analogues of roscovitine **11c** and purvalanol A **13**, elevated temperatures were still required for successful conversion with the corresponding amines (Table 1, compare entries 17 and 18 or 21 and 22). Compound **13**, although isolated as a pure substance via preparative HPLC, exhibited multiple signals in both the ^1^H and ^13^C NMR spectra (in DMSO-*d*_6_ and CDCl_3_), indicative of the presence of tautomeric forms.

In general, a compromise had to be reached in the synthesis of target compounds **11a**–**c** and **13**, balancing both the dominance of the desired product and the chromatographic separability of the reaction mixture.

The use of solvents in the preparation of the *N*7 analogue of olomoucine **11a** (reaction of compound **9** with 2-aminoethanol; Table 1, entries 5–14) proved ineffective. The reactions were carried out in *n*-butanol, anisole, DMSO, and DMF as 1 M solutions at 100–150 °C in a sealed vial. As shown in Table 1, the presence of a solvent had no beneficial effect - it significantly slowed the conversion of the starting material and failed to suppress the formation of the cleavage product. In the case of DMF, a transamination reaction between the solvent and 2-aminoethanol led to the formation of the 2-(dimethylamino) derivative **15**, without, surprisingly, any cleavage of the imidazole ring. Similarly, no ring cleavage was observed upon treatment with sodium methoxide in DMF, where the expected 2-methoxy derivative **16** was obtained (Scheme 6).

**Scheme 6:**
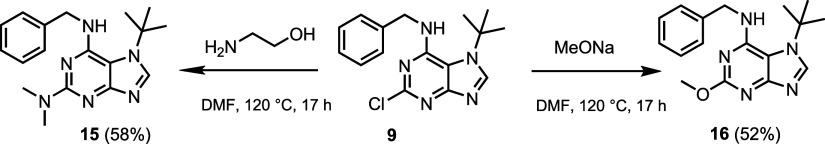
Reaction of **9** with 2-Aminoethanol/DMF and MeONa/DMF

Although the results obtained for compound **15** suggest that imidazole ring cleavage may be specific to primary amines, cleavage was also observed in the case of the secondary amine 2-(methylamino)­ethanol (Scheme 7, eq 5).

**Scheme 7:**
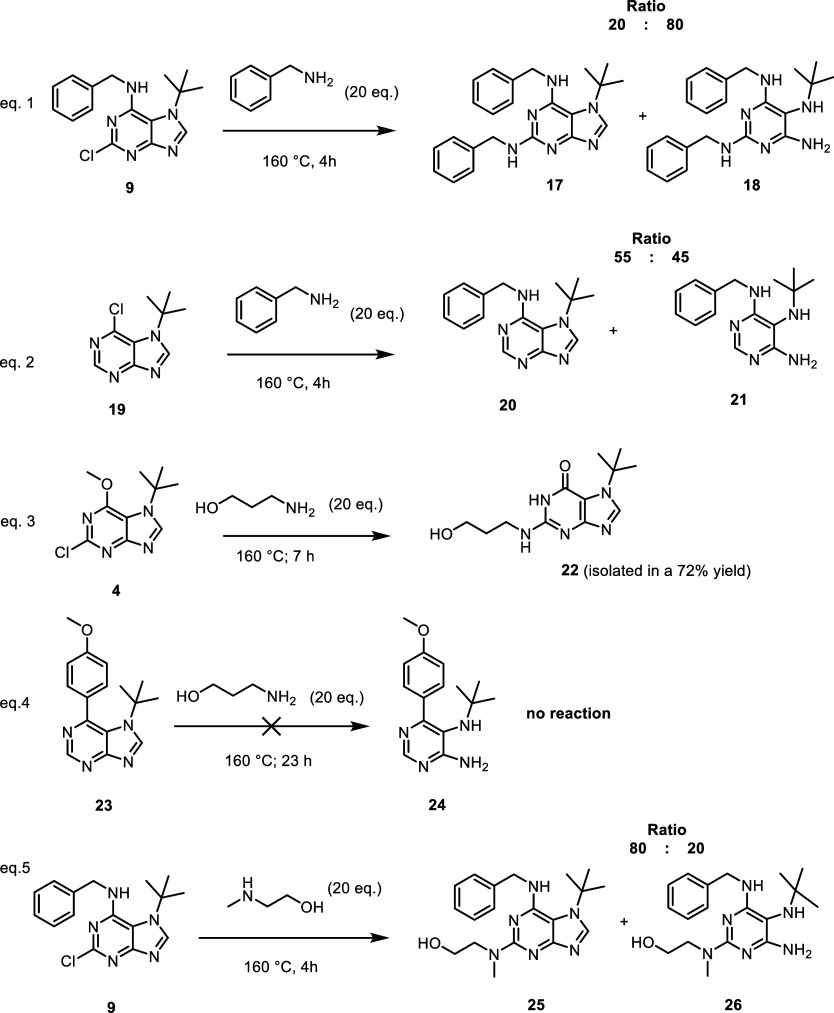
LC/MS Analytical Study of the Stability of the Purine Ring with *N*7 *tert*-Butyl Group

The *tert*-butyl group at the *N*7 position of the purine ring appears to exert a significant influence on the system’s reactivity. Several questions regarding the type of the substitution on the purine ring arose from the experimental observations and were subsequently addressed through the proposed reactions (see Schemes 7 and 8). These reactions were carried out on an analytical scale (∼10 mg) at 160 °C to ensure maximum conversion of the starting materials. The resulting mixtures were subjected to analysis by LC/MS.

**Scheme 8:**
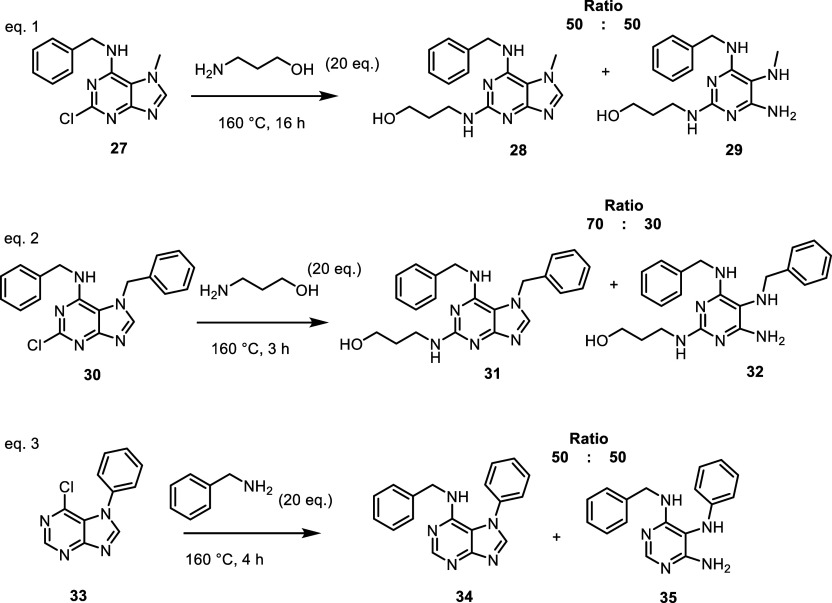
LC/MS Analytical Study of the Stability of the Purine Ring with Various *N*7 Substitutions

Not only amino alcohols but also other primary amines (e.g., benzylamine) were found to induce the opening of the imidazole ring in the *N*7-substituted precursor **9** (Scheme 7, eq 1). Furthermore, we demonstrated that the presence of a chlorine atom at the *C*2 position is not a prerequisite for imidazole ring cleavage. This was evidenced by the reaction of unsubstituted 7-(*tert*-butyl)-6-chloropurine **19** ^14^ with benzylamine, which yielded a mixture of *N*-benzylated products containing both intact purine **20** and ring-opened structure **21** (Scheme 7, eq 2).

To further explore the effect of *C*6 substitution on ring stability, we reacted 3-aminopropanol with two additional derivatives bearing different substituents at the *C*6 position: alkoxy group (compound **4**) and aryl group (compound **23** ^14^). Under these conditions, the purine ring remained stable for both derivatives, even at elevated temperatures (Scheme 7, eqs 3–4). However, in the case of compound **4**, elimination of the methoxy group was observed, leading to the formation of compound **22**, as confirmed by both MS and NMR analysis.

The opening of the imidazole ring upon substitution of the chlorine atom at the *C*2 position with aliphatic amines in *C*6-alkylamino, *N*7-substituted purines—leading to 2,6,7-trisubstituted derivatives—has not been previously described. However, in the literature where the synthesis of such compounds is reported, authors note low yields. For example, the synthesis of isolomoucine afforded only a 28% yield,^31^ and similarly low yields for related substitutions have been reported in other studies.^13^

Based on the above findings, we also investigated whether the nature of the *N*7 substituent affects imidazole ring cleavage in reactions with primary aliphatic amines. To this end, a series of 6-benzylaminopurines bearing representative *N*7 substituents - methyl (compound **27**),^31,32^ benzyl (compound **30**),^33^ and 6-chlorophenyl (compound **33**)^34^ were prepared and subjected to reaction at 160 °C with amines.

The reactions (Scheme 8, eqs 1–3) confirmed that the tested 6-benzylaminopurine derivatives are unstable under these conditions and undergo conversion to a mixture of the corresponding substituted purines and ring-cleaved pyrimidine-diamine derivatives.

We investigated the biological activity of *tert*-butylated purine derivatives. However, preliminary cytotoxicity testing against leukemic cell lines K562 and MV-4-11, as well as CDK2/E kinase inhibition assays, did not show significant activity, with IC_50_ values in the tens of micromolar range.

## Conclusion

The direct SnCl_4_-catalyzed regioselective *tert*-butylation of 2,6-dichloropurine using *t*-BuBr or *t*-BuOAc is described, affording the corresponding *N*7 and *N*9 isomers in good yields. These precursors have been employed for the chemical modification of known purine-based CDK inhibitors; however, the introduction of a *tert*-butyl group did not result in a significant enhancement of biological activity. Interestingly, the presence of a *tert*-butyl group at the *N*7 position of the purine scaffold exhibits distinct chemical properties. It influences the reactivity of the chlorine atom at the *C*6 position, as well as the stability of the imidazole ring during chlorine substitution at the *C*2 position in reactions with amines, compared to the corresponding *N*9 isomers, in which the imidazole ring remains stable. Moreover, these substitution reactions with amines were found to be temperature-dependent and affected not only by the *tert*-butyl group, but also by other substituents located at the *N*7 position. These results reveal a previously unrecognized trend relevant to the synthesis of underexplored 2,6,7-trisubstituted purines.

## Experimental Section

### General Methods

All reagents were purchased from commercial suppliers and used without purification. Solvents were dried according to standard procedures and stored with molecular sieve 3A. Reactions were monitored by the LC/MS analyses using a UPLC Waters Acquity system equipped with PDA and QDa detectors. The system contained an XSelect HSS T3 (Waters) 3 mm × 50 mm C18 reverse phase column XP (2.5 μm particles). Mobile phases: 10 mM ammonium acetate in HPLC grade water (A) and gradient grade ACN for HPLC (B). A gradient was mainly formed from 20% to 80% of B in 4.5 min and kept for 1 min, with a flow rate of 0.6 mL/min. The MS ESI operated at a 25 V cone voltage, 600 °C probe temperature, and 120 °C source temperature. ^1^H and ^13^C NMR spectra were measured on JEOL ECA 400II NMR spectrometer (^1^H: 399.78 MHz, ^13^C: 100.53 MHz). Chemical shifts (δ) are reported in ppm and referenced to the middle peak of the solvent signal (CDCl_3_: 7.26 ppm, 77.00 ppm, DMSO-*d*_6_: 2.49 ppm, 39.5 ppm). High resolution mass spectra (HRMS) measurements were performed on UPLC Dionex Ultimate 3000 equipped with an Orbitrap Elite high-resolution mass spectrometer, Thermo Exactive plus. Purification was carried out using semipreparative HPLC Agilent on a YMC-Actus Pro 20 mm × 150 mm C18 reversed-phase column (5 μm particles). Mobile phases: 10 mM aqueous ammonium acetate and gradient grade ACN for HPLC at a flow rate of 15 mL/min. All microwave reactions were carried out in a CEM-Discover microwave reactor operating at 2.45 GHz with continuous irradiation of 300W maximum power. The microwave irradiation of required power was used. Once the desired temperature was reached, the mixture was held at this temperature for given time. Thin layer chromatography (TLC) was performed on precoated silica gel 60 F254 plates and visualized by exposure to UV light (254 or 366 nm). Column chromatography was carried out by using silica gel grade 60, meshes size 230 to 400 Å. Melting points were measured on Boetius stage apparatus and are uncorrected.

#### 7-(*tert*-Butyl)-2,6-dichloro-7*H*-purine (**2**)

**Method A** (with *tert*-butyl bromide): To a suspension of 2,6-dichloropurine (**1**) (5.0 g, 26.5 mmol) in anhydrous acetonitrile (190 mL) was added *N*,*O*-*bis*(trimethylsilyl)­acetamide (9.7 mL, 39.7 mmol) under argon. This mixture was refluxed for 30 min to obtain a clear solution. After cooling in an ice bath to 0 °C, SnCl_4_ (6.5 mL, 55.6 mmol) and then *tert*-butyl bromide (8.9 mL, 79.4 mmol) were added. The mixture was stirred **at 0 °C** for 3 h. Afterward, the reaction mixture was quenched with isopropanol (53 mL), diluted with chloroform (265 mL), washed with water (4 × 200 mL), and brine (370 mL). The organic phase was dried (MgSO_4_), and the solvents were evaporated under reduced pressure to obtain a yellowish crystalline compound as a crude product of sufficient purity (94% based on ^1^H NMR). The yield was 4.68 g (72%). The final product was obtained by crystallization from isopropanol (335 mL). The suspension was heated to dissolve the compound, then cooled in an ice bath to induce crystallization. The resulting solid was collected by filtration, washed with isopropanol, and dried at 120 °C for 1 h, affording a white crystalline compound (3.91 g) with a melting point of 193–195 °C (200–205 °C^15^). ^1^H NMR (400 MHz, CDCl_3_): δ 8.47 (s, 1H), 1.90 (s, 9H). ^13^C­{^1^H} NMR (101 MHz, CDCl_3_): δ 165.5 (C-4), 152.8 (C-2), 148.3 (C-8), 143.6 (C-6), **122.4 (C-5)**, 59.6 (C), 31.1 (CH_3_). HRMS (ESI, *m*/*z*): [M + H]^+^ calcd for C_9_H_11_Cl_2_N_4_, 245.0355; found, 245.0364.

### **Method B** (with *tert*-Butyl Acetate)

The title compound **2** was prepared in a similar manner as purine **2** (Method A) using 2,6-dichloropurine (189 mg, 1.0 mmol), *N*,*O*-*bis*(trimethylsilyl)­acetamide (368 μL, 1.5 mmol), SnCl_4_ (248 μL, 2.1 mmol and *tert*-butyl acetate (404 μL, 3.0 mmol) in acetonitrile (8 mL). The resulting reaction mixture was processed with a proportional amount of auxiliary reagents. After removal of the solvents under reduced pressure and drying of the solid at 120 °C for 1 h, the crude product of sufficient purity was obtained, yielding 157 mg (64%). This method afforded compound **2** with the same spectral characteristics as obtained by Method A.

#### 9-(*tert*-Butyl)-2,6-dichloro-9*H*-purine (**3**)

**Method A** (with *tert*-butyl bromide): To a suspension of 2,6-dichloropurine (**1**) (5.0 g, 26.5 mmol) in anhydrous acetonitrile (190 mL) was added *N*,*O*-*bis*(trimethylsilyl)­acetamide (9.7 mL, 39.7 mmol) under argon. This mixture was refluxed for 30 min to obtain a clear solution. After cooling in an ice bath to 0 °C, SnCl_4_ (6.5 mL, 55.6 mmol) and then *tert*-butyl bromide (8.9 mL, 79.4 mmol) were added. The mixture was **refluxed** for 3 h. After cooling, the reaction mixture was quenched with isopropanol (53 mL), diluted with chloroform (265 mL), washed with water (4 × 200 mL), and brine (370 mL). The organic phase was dried (MgSO_4_), and the solvents were evaporated under reduced pressure to obtain a yellowish crystalline compound as a crude product of sufficient purity (96% based on ^1^H NMR). The yield was 4.88 g (75%). The final product was obtained by crystallization from isopropanol (100 mL). The suspension was heated to dissolve the compound, then cooled in an ice bath to induce crystallization. The resulting solid was collected by filtration, washed with isopropanol, and dried at 120 °C for 1 h, affording a white crystalline compound (3.69 g) with a melting point of 181–182 °C (175–180 °C^15^). ^1^H NMR (400 MHz, CDCl_3_): δ 8.17 (s, 1H), 1.81 (s, 9H). ^13^C­{^1^H} NMR (101 MHz, CDCl_3_): δ 153.1 (C-2), 151.8 (C-4), 151.7 (C-6), 143.6 (C-8), **131.9 (C-5)**, 59.0 (C), 28.9 (CH_3_). HRMS (ESI, *m*/*z*): [M + H]^+^ calcd for C_9_H_11_Cl_2_N_4_, 245.0355; found, 245.0357.

### **Method B** (with *tert*-Butyl Acetate)

The title compound **3** was prepared in a similar manner as purine **3** (Method A) using 2,6-dichloropurine (189 mg, 1.0 mmol), *N*,*O*-*bis*(trimethylsilyl)­acetamide (368 μL, 1.5 mmol), SnCl_4_ (248 μL, 2.1 mmol) and *tert*-butyl acetate (404 μL, 3.0 mmol) in acetonitrile (8 mL). The resulting reaction mixture was processed with a proportional amount of auxiliary reagents. After removal of the solvents under reduced pressure and drying of the solid at 120 °C for 1 h, the crude product of sufficient purity was obtained, yielding 197 mg (80%). This method afforded compound **3** with the same spectral characteristics as obtained by Method A.

#### 7-(*tert*-Butyl)-2-chloro-6-methoxy-7*H*-purine (**4**)

To a mixture of 7-(*tert*-butyl)-2,6-dichloropurine (**2**) (100 mg, 0.41 mmol) in anhydrous methanol (2 mL) was added a solution of sodium methoxide in methanol (1.22 mL, 1 M solution). This mixture was stirred for 30 min at room temperature and then neutralized with diluted acetic acid, and toluene (4 mL) was added. The organic phase was washed with water (4 × 2 mL), brine (2 mL), dried (MgSO_4_) and evaporated under reduced pressure to obtain a white crystalline solid. The yield was 68.5 mg (70%) as a crude product of sufficient purity. ^1^H NMR (400 MHz, CDCl_3_): δ 8.16 (s, 1H), 4.19 (s, 3H), 1.72 (s, 9H). ^13^C­{^1^H} NMR (101 MHz, CDCl_3_): δ 164.8, 156.3, 152.5, 144.3, 111.5, 58.3, 55.0, 30.0. HRMS (ESI, *m*/*z*): [M + H]^+^ calcd for C_10_H_13_ClN_4_O, 241.0853; found, 241.0853.

#### *N*-Benzyl-9-(*tert*-butyl)-2-chloro-9*H*-purin-6-Amine (**5**)

A mixture of 9-(*tert*-butyl)-2,6-dichloropurine (**3**) (1.0 g, 4.1 mmol), DIPEA (1.42 mL, 8.2 mmol), and benzylamine (0.67 mL, 6.12 mmol) in *n*-butanol (21 mL) was heated at 110 °C under argon for 2 h. The resulting solution was gradually cooled in an ice bath to induce crystallization. The crystalline solid was collected by filtration, washed with isopropanol, and dried at 80 °C for 1 h, affording a white crystalline compound. The yield was 982 mg (72%), mp 170–172 °C. ^1^H NMR (400 MHz, CDCl_3_): δ 7.63 (s, 1H), 7.37–7.27 (m, 5H), 6.76 (br s, 1H), 4.83 (br s, 2H), 1.73 (s, 9H). ^13^C­{^1^H} NMR (101 MHz, CDCl_3_): δ 155.2, 153.5, 150.4, 138.1, 137.7, 128.7, 127.9, 127.6, 119.7, 57.6, 44.5, 29.0. HRMS (ESI, *m*/*z*): [M + H]^+^ calcd for C_16_H_18_ClN_5_, 316.1323; found, 316.1327.

#### 9-(*tert*-Butyl)-2-chloro-*N*-(3-chlorophenyl)-9*H*-purin-6-amine (**6**)

A mixture of 9-(*tert*-butyl)-2,6-dichloropurine (**3**) (245 mg, 1.0 mmol), DIPEA (280 μL, 1.6 mmol), and 3-chloroaniline (127 μL, 1.2 mmol) in *n*-butanol (1.5 mL) was heated in a sealed vial at 120 °C under argon for 6 h. The resulting mixture was concentrated under vacuum, and the residue was crystallized from an isopropanol-water mixture (5:2, v/v; 7 mL). The crystalline solid was collected by filtration, washed with water, and dried at 80 °C for 1 h, affording a brownish crystalline compound. The yield was 168 mg (50%), mp 109–112 °C. ^1^H NMR (400 MHz, CDCl_3_): δ 8.09 (s, 1H), 7.87 (s, 1H), 7.86 (t, *J* = 2.0 Hz, 1H), 7.68 (dd, *J* = 8.1, 2.0 Hz, 1H), 7.29 (t, *J* = 8.1 Hz, 1H), 7.08 (dd, *J* = 7.9, 1.8 Hz, 1H), 1.79 (s, 9H). ^13^C­{^1^H} NMR (101 MHz, CDCl_3_): δ 152.8, 152.2, 151.0, 139.5, 138.9, 134.6, 130.0, 123.7, 120.6, 120.0, 118.0, 58.0, 29.1. HRMS (ESI, *m*/*z*): [M + H]^+^ calcd for C_15_H_15_Cl_2_N_5_, 336.0778; found, 336.0783.

#### 2-((6-(Benzylamino)-9-(*tert*-butyl)-9*H*-purin-2-yl)­amino)­ethan-1-ol (**7a**)

A mixture of *N*9 precursor **5** (100 mg, 0.32 mmol) in 2-aminoethan-1-ol (153 μL, 2.53 mmol) was heated in a sealed vial at 150 °C for 3 h. After cooling, the reaction mixture was diluted with chloroform (5 mL), washed with water (4 × 2 mL), and brine (2 mL). After verifying that the washing liquids have a neutral pH, the organic phase was dried (MgSO_4_), and the solvent was evaporated under reduced pressure to obtain a yellowish solid. The yield was 96.5 mg (89%). The product was crystallized from hot acetonitrile (2.2 mL), yielding 37 mg of a white crystalline compound, mp 116–118 °C. ^1^H NMR (400 MHz, CDCl_3_): δ 7.42 (s, 1H), 7.34–7.20 (m, 5H), 6.39 (br s, 1H), 5.27 (t, *J* = 5.8 Hz, 1H), 4.73 (d, *J* = 4.3 Hz, 2H), 4.45–4.67 (br s, 1H), 3.79 (t, *J* = 4.9 Hz, 2H), 3.53 (q, *J* = 5.6 Hz, 2H), 1.67 (s, 9H). ^13^C­{^1^H} NMR (101 MHz, CDCl_3_): δ 159.5, 155.0, 151.0, 139.0, 134.7, 128.5, 127.6, 127.2, 115.8, 64.1, 56.4, 45.0, 44.3, 28.9. HRMS (ESI, *m*/*z*): [M + H]^+^ calcd for C_18_H_24_N_6_O, 341.2084; found, 341.2086.

#### 3-((6-(Benzylamino)-9-(*tert*-butyl)-9*H*-purin-2-yl)­amino)­propan-1-ol (**7b**)

This compound was prepared in a similar manner as compound **7a** using *N*9 precursor **5** (100 mg, 0.32 mmol) and 3-aminopropan-1-ol (194 μL, 2.54 mmol). After evaporation of the processing solvent, the semisolid compound was purified by column chromatography (silica gel, 10 × 4 cm i.d., CHCl_3_–MeOH, 40:1, v/v). The yield was 83 mg, (74%) as a white solid. ^1^H NMR (400 MHz, CDCl_3_): δ 7.46 (s, 1H), 7.36–7.22 (m, 5H), 6.19 (br s, 1H), 4.95 (t, *J* = 6.3 Hz, 1H), 4.75 (d, *J* = 4.3 Hz, 2H), 3.70 (t, *J* = 5.8 Hz, 2H), 3.58 (q, *J* = 6.3 Hz, 2H), 1.78 (quin, *J* = 6.2 Hz, 2H), 1.70 (s, 9H). ^13^C­{^1^H} NMR (101 MHz, CDCl_3_): δ 159.3, 155.0, 151.4, 138.9, 134.7, 128.5, 127.7, 127.2, 115.5, 59.3, 56.4, 44.4, 38.0, 33.0, 29.0. HRMS (ESI, *m*/*z*): [M + H]^+^ calcd for C_19_H_26_N_6_O, 355.2241; found, 355.2233.

#### (*R*)-2-((6-(Benzylamino)-9-(*tert*-butyl)-9*H*-purin-2-yl)­amino)­butan-1-ol (**7c**)

This compound was prepared in a similar manner as compound **7a** using *N*9 precursor **5** (100 mg, 0.32 mmol) and (*R*)-2-aminobutan-1-ol (242 μL, 2.56 mmol). To achieve full conversion, the reaction mixture was heated at 150 °C for 30 h. After evaporation of the processing solvent, the resulting crude solid (104 mg, 89%) was purified by crystallization from hot acetonitrile (2 mL), yielding a white crystalline compound (67 mg), mp 132–134 °C. ^1^H NMR (400 MHz, CDCl_3_): δ 7.45 (s, 1H), 7.36–7.23 (m, 5H), 6.18 (br s, 1H), 4.87 (d, *J* = 6.2 Hz, 1H), 4.78–4.68 (m, 2H), 4.44 (br s, 1H), 3.89 (quind, *J* = 7.0, 3.1 Hz, 1H), 3.82 (dd, *J* = 10.7, 3.1 Hz, 1H), 3.62 (dd, *J* = 10.7, 7.6 Hz, 1H), 1.69 (s, 9*H*), 1.58 (decaplet, *J* = 7.3 Hz, 2H), 1.00 (t, *J* = 7.5 Hz, 3H). ^13^C­{^1^H} NMR (101 MHz, CDCl_3_): δ 159.3, 154.9, 150.9, 138.9, 134.8, 128.5, 127.7, 127.3, 115.8, 67.7, 56.4, 55.9, 44.4, 28.9, 24.9, 10.9. HRMS (ESI, *m*/*z*): [M + H]^+^ calcd for C_20_H_28_N_6_O, 369.2397; found, 369.2391.

#### (*R*)-2-((9-(*tert*-Butyl)-6-((3-chlorophenyl)­amino)-9*H*-purin-2-yl)­amino-3-methylbutan-1-ol (**8**)

A mixture of *N*9 precursor **6** (148 mg, 0.44 mmol) in (*R*)-2-amino-3-methylbutan-1-ol (392 μL; 3.52 mmol) was heated in a sealed vial at 150 °C for 22 h. After cooling, the reaction mixture was diluted with chloroform (10 mL), washed with water (4 × 8 mL), and brine (10 mL). After verifying that the washing liquids have a neutral pH, the organic phase was dried (MgSO_4_), and the solvent was evaporated under reduced pressure to obtain a yellowish solid. The yield was 136 mg (77%). The product was crystallized from a hot acetonitrile–water mixture (1:1, v/v), filtered off, and dried at 80 °C for 1 h, yielding 90 mg of a white crystalline compound, mp 144–146 °C. ^1^H NMR (400 MHz, CDCl_3_): δ 7.97 (s, 1H), 7.90 (s, 1H), 7.57 (s, 1H), 7.46 (d, *J* = 8.2 Hz, 1H), 7.19 (t, *J* = 8.1 Hz, 1H), 6.98 (dd, *J* = 7.9, 0.9 Hz, 1H), 5.04 (d, *J* = 7.9 Hz, 1H), 4.00–3.94 (m, 1H), 3.91 (d, *J* = 10.4 Hz, 1H), 3.81 (br s, 1H), 3.75 (dd, *J* = 10.2, 7.5 Hz, 1H), 2.02 (td, *J* = 13.2, 7.0 Hz, 1H), 1.69 (s, 9H), 1.04 (d, *J* = 6.7 Hz, 6H). ^13^C­{^1^H} NMR (101 MHz, CDCl_3_): δ 159.1, 152.1, 151.6, 140.6, 135.5, 134.3, 129.7, 122.5, 119.7, 117.6, 116.1, 64.6, 59.3, 56.6, 29.9, 28.9, 19.5, 19.1. HRMS (ESI, *m*/*z*): [M + H]^+^ calcd for C_20_H_28_ClN_6_O, 403.2008; found, 403.2003.

#### *N*-Benzyl-7-(*tert*-butyl)-2-chloro-7*H*-purin-6-amine (**9**)

A mixture of 7-(*tert*-butyl)-2,6-dichloropurine (**2**) (1.0 g, 4.1 mmol), DIPEA (1.42 mL, 8.2 mmol), and benzylamine (0.67 mL, 6.12 mmol) in *n*-butanol (21 mL) was heated at 110 °C under argon for 3 h. The resulting solution was gradually cooled in an ice bath to induce crystallization. The crystalline solid was collected by filtration, washed with isopropanol, and dried at 80 °C for 1 h, affording a white crystalline compound. The yield was 894 mg (69%), mp 190–191 °C. ^1^H NMR (400 MHz, CDCl_3_): δ 8.10 (s, 1H), 7.39–7.31 (m, 5H), 5.49 (br s, 1H), 4.85 (d, *J* = 5.0 Hz, 2H), 1.73 (s, 9H). ^13^C­{^1^H} NMR (101 MHz, CDCl_3_): δ 163.1, 154.1, 150.2, 142.9, 137.6, 129.0, 127.9 (2 x C), 110.7, 56.5, 46.3, 31.6. HRMS (ESI, *m*/*z*): [M + H]^+^ calcd for C_16_H_18_ClN_5_, 316.1323; found, 316.1327.

#### 7-(*tert*-Butyl)-2-chloro-*N*-(3-chlorophenyl)-7*H*-purin-6-amine (**10**)

To a solution of *N*-(3-chlorophenyl)­formamide (317 mg, 2.04 mmol) in anhydrous DMSO (4 mL) was added sodium hydride (60% dispersion in mineral oil, 207 mg, 5.2 mmol). The suspension was stirred at room temperature for 20 min under argon in a sealed vial. Then, 7-(*tert*-butyl)-2,6-dichloropurine (**2**) (500 mg, 2.04 mmol) was added, and the resulting mixture was heated with stirring at 80 °C for 1 h. After cooling, water (8 mL) was added, and stirring was continued for an additional 1 h. The precipitated compound was filtered off, washed with water, and dried to afford an impure solid (460 mg). This product was recrystallized from hot acetonitrile (8 mL) to give a light green crystalline compound. The yield was 261 mg (38%), mp 221–222 °C. ^1^H NMR (400 MHz, DMSO-*d*_6_): δ 8.59 (s, 1H), 7.99 (s, 1H), 7.62 (s, 1H), 7.47–7.39 (m, 2H), 7.24–7.22 (m, 1H), 1.78 (s, 9H). ^13^C­{^1^H} NMR (101 MHz, DMSO-*d*_6_): δ 163.8, 151.6, 148.6, 146.3, 140.2, 132.7, 130.0, 124.2, 123.4, 122.4, 111.8, 57.6, 31.0. HRMS (ESI, *m*/*z*): [M + H]^+^ calcd for C_15_H_15_Cl_2_N_5_, 336.0778; found, 336.0783.

#### 2-((6-(Benzylamino)-7-(*tert*-butyl)-7*H*-purin-2-yl)­amino)­ethan-1-ol (**11a**)

A mixture of *N*7 precursor **9** (100 mg, 0.32 mmol) in 2-aminoethan-1-ol (383 μL, 6.33 mmol) was heated in a sealed vial at 100 °C for 11 h. After cooling, the reaction mixture was diluted with chloroform (2 mL), washed with water (4 × 2 mL), and brine (2 mL). After verifying that the washing liquids have a neutral pH, the organic phase was dried (MgSO_4_), and the solvent was evaporated under reduced pressure to obtain an oily product, which was purified by preparative HPLC. The yield was 58 mg (54%) as a white solid. ^1^H NMR (400 MHz, DMSO-*d*_6_): δ 8.01 (s, 1H), 7.37–7.17 (m, 5H), 6.39 (t, *J* = 5.2 Hz, 1H), 5.90 (t, *J* = 5.8 Hz, 1H), 4.76 (d, *J* = 5.5 Hz, 2H), 3.44 (t, *J* = 6.0 Hz, 2H), 3.25 (q, *J* = 5.9 Hz, 2H), 1.68 (s, 9H). ^13^C­{^1^H} NMR (101 MHz, DMSO-*d*_6_): δ 163.4, 158.5, 149.4, 141.7, 140.6, 128.1, 127.1, 126.3, 105.5, 60.6, 55.6, 44.1, 43.8, 30.9. HRMS (ESI, *m*/*z*): [M + H]^+^ calcd for C_18_H_24_N_6_O, 341.2084; found, 341.2082.

#### 3-((6-(Benzylamino)-7-(*tert*-butyl)-7*H*-purin-2-yl)­amino)­propan-1-ol (**11b**)

This compound was prepared in a similar manner as compound **11a** using *N*7 precursor **9** (100 mg, 0.32 mmol) and 3-aminopropan-1-ol (484 μL, 6.33 mmol). The reaction mixture was heated at 100 °C for 14 h. After evaporation of the processing solvent, the resulting oily product was purified by preparative HPLC. The yield was 54 mg (48%) of a white solid. ^1^H NMR (400 MHz, DMSO-*d*_6_): δ 8.00 (s, 1H), 7.38–7.16 (m, 5H), 6.38 (br s, 1H), 6.06 (t, *J* = 6.2 Hz, 1H), 4.78 (d, *J* = 4.9 Hz, 2H), 3.41 (t, *J* = 6.3 Hz, 2H), 3.23 (q, *J* = 6.4 Hz, 2H), 1.68 (s, 9H), 1.58 (quin, *J* = 6.4 Hz, 2H). ^13^C­{^1^H} NMR (101 MHz, DMSO-*d*_6_): δ 163.5, 158.6, 149.4, 141.6, 140.7, 128.1, 127.1, 126.3, 105.3, 58.7, 55.6, 44.1, 38.1, 32.8, 30.9. HRMS (ESI, *m*/*z*): [M + H]^+^ calcd for C_19_H_26_N_6_O, 355.2241; found, 355.2273.

#### (*R*)-2-((6-(Benzylamino)-7-(*tert*-butyl)-7*H*-purin-2-yl)­amino)­butan-1-ol (**11c**)

This compound was prepared in a similar manner as compound **11a** using *N*7 precursor **9** (100 mg, 0.32 mmol) and (*R*)-2-aminobutan-1-ol (598 μL, 6.33 mmol). The reaction mixture was heated at 130 °C for 6 h. After evaporation of the processing solvent, the resulting oily product was purified by preparative HPLC. The yield was 37 mg (31%) of a white solid. ^1^H NMR (400 MHz, DMSO-*d*_6_): δ 8.00 (s, 1H), 7.37–7.16 (m, 5H), 6.36 (br s, 1H), 5.59 (d, *J* = 8.2 Hz, 1H), 4.77 (ddd, *J* = 24.9, 15.0, 5.3 Hz, 2H), 3.72 (td, *J* = 13.3, 5.4 Hz, 1H), 3.42 (dd, *J* = 10.5, 4.7 Hz, 1H), 3.29 (dd, *J* = 10.5, 5.6 Hz, 1H), 1.68 (s, 9H), 1.59–1.52 (m, 1H), 1.43–1.32 (m, 1H), 0.78 (t, *J* = 7.3 Hz, 3H). ^13^C­{^1^H} NMR (101 MHz, DMSO-*d*_6_): δ 163.2, 158.4, 149.4, 141.7, 140.6, 128.1, 127.1, 126.3, 105.3, 63.2, 55.7, 53.8, 44.1, 30.9, 23.8, 10.5. HRMS (ESI, *m*/*z*): [M + H]^+^ calcd for C_20_H_28_N_6_O, 369.2397; found, 369.2393.

#### 2-((4-Amino-6-(benzylamino)-5-(*tert*-butylamino)­pyrimidin-2-yl)­amino)­ethan-1-ol (**12a**)

This compound was prepared in a similar manner as compound **11a** using *N*7 precursor **9** (100 mg, 0.32 mmol) and 2-aminoethan-1-ol (153 μL, 2.53 mmol). The reaction mixture was heated at 100 °C for 29 h. After evaporation of the processing solvent, the resulting oily product was purified by preparative HPLC. The yield was 62 mg (59%) of a pink colored semisolid product (air unstable). ^1^H NMR (400 MHz, CDCl_3_): δ 7.32–7.25 (m, 5H), 5.94 (t, *J* = 5.2 Hz, 1H), 5.51 (br s, 1H), 4.55 (d, *J* = 5.8 Hz, 2H), 3.74 (t, *J* = 4.7 Hz, 2H), 3.47 (q, *J* = 4.6 Hz, 2H), 1.13 (s, 9H). ^13^C­{^1^H} NMR (101 MHz, CDCl_3_): δ 162.7, 154.4, 153.1, 138.5, 128.6, 127.5, 127.4, 92.7, 61.8, 54.6, 45.0, 44.1, 30.6. HRMS (ESI, *m*/*z*): [M + H]^+^ calcd for C_17_H_26_N_6_O, 331.2241; found, 331.2236.

#### 3-((4-Amino-6-(benzylamino)-5-(*tert*-butylamino)­pyrimidin-2-yl)­amino)­propan-1-ol (**12b**)

This compound was prepared in a similar manner as compound **11a** using N7 precursor **9** (100 mg, 0.32 mmol) and 3-aminopropan-1-ol (194 μL, 2.53 mmol). The reaction mixture was heated at 100 °C for 29 h. After evaporation of the processing solvent, the resulting oily product was purified by preparative HPLC. The yield was 57 mg (52%) of a light red colored product (air unstable). ^1^H NMR (400 MHz, CDCl_3_): δ 7.32–7.25 (m, 5H), 5.94 (t, *J* = 5.2 Hz, 1H), 5.51 (br s, 1H), 4.55 (d, *J* = 5.8 Hz, 2H), 3.74 (t, *J* = 4.7 Hz, 2H), 3.47 (q, *J* = 4.6 Hz, 2H), 1.13 (s, 9H). ^13^C­{^1^H} NMR (101 MHz, CDCl_3_): δ 162.4, 160.6, 159.2, 139.7, 128.5, 127.6, 127.1, 94.8, 58.1, 54.3, 45.0, 37.0, 33.6, 30.9. HRMS (ESI, *m*/*z*): [M + H]^+^ calcd for C_18_H_26_N_6_O, 345.2397; found, 345.2397.

#### (*R*)-2-((7-(*tert*-Butyl)-6-((3-chlorophenyl)­amino)-7*H*-purin-2-yl)­amino-3-methylbutan-1-ol (**13**)

This compound was prepared in a similar manner as compound **11a** using *N*7 precursor **10** (100 mg, 0.3 mmol) and (*R*)-2-amino-3-methylbutan-1-ol (663 μL, 6 mmol). The reaction mixture was heated at 120 °C for 6 h. After evaporation of the processing solvent, the resulting oily product was purified by preparative HPLC. The yield was 29 mg (24%) of a white solid. The spectral data indicate a mixture of tautomers: ^1^H NMR (400 MHz, DMSO-*d*_6_): δ 9.19 (s, 1H), 8.19 (s, 1H), 7.86 (s, 1H), 7.64 (s, 1H), 7.46 (s, 2H), 7.32 (dd, *J* = 18.2, 8.1 Hz, 2H), 7.04 (t, *J* = 6.6 Hz, 2H), 6.86 (s, 1H), 6.80 (d, *J* = 7.9 Hz, 1H), 6.54 (d, *J* = 8.5 Hz, 1H), 5.95 (d, *J* = 8.9 Hz, 1H), 4.67 (s, 1H), 4.46 (s, 1H), 3.72–3.65 (m, 2H), 3.47–3.37 (m, 4H), 1.87–1.80 (m, 2H), 1.73 (s, 18H), 0.88–0.83 (m, 12H). ^13^C­{^1^H} NMR (101 MHz, DMSO-*d*_6_): δ 165.0, 158.7, 158.2, 151.1, 150.3, 147.1, 144.3, 141.9, 140.2, 139.6, 133.8, 132.8, 131.1, 130.0, 121.8, 121.6, 121.5, 120.6, 120.2, 119.4, 108.2, 107.3, 61.3, 60.7, 57.6, 57.4, 56.6, 56.3, 30.9, 29.6, 28.6, 28.1, 19.4, 19.3, 18.8, 18.4. HRMS (ESI, *m*/*z*): [M + H]^+^ calcd for C_20_H_27_ClN_6_O, 403.2008; found, 403.2004.
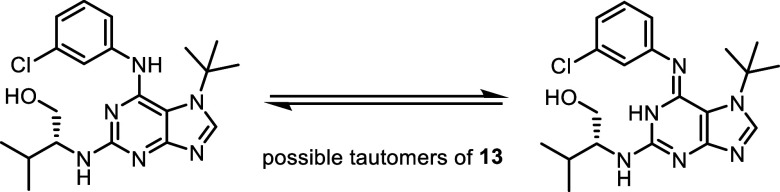


#### (*R*)-2-((4-Amino-5-(*tert*-butylamino)-6-((3-chlorophenyl)­amino)­pyrimidin-2-yl)­amino)-3-methylbutan-1-ol (**14**)

This compound was prepared in a similar manner as compound **13** using *N*7 precursor **10** (50 mg, 0.15 mmol) and (*R*)-2-amino-3-methylbutan-1-ol (663 μL, 6 mmol). The reaction mixture was heated at 160 °C for 6 h. After evaporation of the processing solvent, the resulting oily product was purified by preparative HPLC. The yield was 25 mg (43%) of a light red colored product (air unstable). ^1^H NMR (400 MHz, CDCl_3_): δ 7.99 (br s, 1H), 7.84 (s, 1H), 7.34–7.28 (m, 1H), 7.15 (t, *J* = 7.9 Hz, 1H), 6.93 (d, *J* = 7.9 Hz, 1H), 5.23 (br s, 2H), 3.84–3.81 (m, 2H), 3.71–3.67 (m, 1H), 1.98–1.92 (m, 1H), 1.18 (s, 9H), 1.00 (s, 6H). ^13^C­{^1^H} NMR (101 MHz, CDCl_3_): δ 160.8, 159.7, 158.8, 141.3, 134.4, 129.6, 121.4, 118.8, 116.7, 96.0, 59.1, 54.4, 30.8, 29.9, 29.7, 19.2, 19.1. HRMS (ESI, *m*/*z*): [M + H]^+^ calcd for C_19_H_29_ClN_6_O, 393.2164; found, 393.2163.

#### *N* ^6^–Benzyl-7-(*tert*-butyl)-*N* ^2^,*N* ^2^-dimethyl-7*H*-purine-2,6-diamine (**15**)

To a solution of *N*7 precursor **9** (75 mg, 0.24 mmol) in anhydrous DMF (1.5 mL), 2-aminoethanol (116 μL, 1.9 mmol) was added. The reaction mixture was then heated in a sealed vial at 120 °C for 17 h in an oil bath. Upon completion, the mixture was subjected to extraction. Ethyl acetate (6 mL) was added, and the organic phase was extracted four times with distilled water (3 mL each) and washed with brine (3 mL). The organic layer was dried over anhydrous MgSO_4_ and concentrated under reduced pressure. The resulting residue was dried at room temperature to yield 45 mg (58%) a light red, powdery crude product. ^1^H NMR (400 MHz, CDCl_3_): δ 7.87 (s, 1H), 7.38–7.28 (m, 5H), 5.15 (t, *J* = 5.3 Hz, 1H), 4.83 (d, *J* = 5.3 Hz, 2H), 3.16 (s, 6H), 1.69 (s, 9H). ^13^C­{^1^H} NMR (101 MHz, CDCl_3_): δ 164.5, 159.6, 149.3, 141.0, 139.2, 128.7, 127.5, 127.3, 105.2, 55.3, 45.6, 37.4, 31.4 HRMS (ESI, *m*/*z*): [M + H]^+^ calcd for C_18_H_25_N_6_, 325.2135; found, 325.2163.

#### *N*-Benzyl-7-(*tert*-butyl)-2-methoxy-7*H*-purin-6-amine (**16**)

Sodium methoxide was prepared by dissolving Na (44 mg, 1.92 mmol) in anhydrous MeOH (1 mL), followed by evaporation to dryness. The resulting solid was dissolved in anhydrous DMF (1.5 mL), and *N*7 precursor **9** (100 mg, 0.32 mmol) was added. The reaction mixture was then heated in a sealed vial at 120 °C for 17 h in an oil bath. Upon completion, the mixture was subjected to extraction. Ethyl acetate (8 mL) was added, and the organic phase was extracted four times with water (4 mL each) and washed with brine (4 mL). The organic layer was dried over anhydrous MgSO_4_ and concentrated under reduced pressure. The resulting residue was crystallized from of ethyl acetate (2.5 mL) and dried at 80 °C for 1 h to afford 51 mg (52%) of a white crystalline product, mp 182–184 °C. ^1^H NMR (400 MHz, CDCl_3_): δ 7.98 (s, 1H), 7.38–7.28 (m, 5H), 5.25 (t, *J* = 4.9 Hz, 1H), 4.84 (d, *J* = 4.9 Hz, 2H), 4.02 (s, 3H), 1.70 (s, 9H). ^13^C­{^1^H} NMR (101 MHz, CDCl_3_): δ 163.8, 162.0, 150.6, 141.8, 138.1, 128.9, 127.7, 127.7, 108.1, 55.9, 54.5, 46.0, 31.5. HRMS (ESI, *m*/*z*): [M + H]^+^ calcd for C_17_H_22_N_5_O, 312.1819; found, 312.1822. Alternatively, this reaction can be performed faster using compound **9** (100 mg) in the absence of DMF, by employing a solution of sodium methoxide (prepared by dissolving 45 mg of sodium in 1 mL of anhydrous methanol) under pressure in a sealed vial at 92 °C for 6 h, resulting in complete conversion of the starting material to product **16**. After analogous workup and crystallization of the crude solid from ethyl acetate (3.5 mL), a higher yield of 68% (67 mg) was obtained.

#### 7-(*tert*-Butyl)-2-((3-hydroxypropyl)­amino)-1,7-dihydro-6*H*-purin-6-one (**22**)

A mixture of the methoxy derivative **4** (68 mg; 0.28 mmol) in 3-aminopropan-1-ol (416 μL; 5.63 mmol) was heated at 160 °C for 7 h under inert conditions in a sealed vial. After cooling, water (10 mL) was added, and the pH was adjusted to neutral with acetic acid. The aqueous phase was extracted with EtOAc (6 × 20 mL), and the combined organic layers were washed with brine (20 mL), dried over MgSO_4_, and concentrated under reduced pressure to yield a yellowish crude solid (66 mg). Purification by column chromatography (silica gel, 10 × 4 cm i.d., CHCl_3_–MeOH, 40:5, v/v) afforded a white solid after evaporation (54 mg; 72%). ^1^H NMR (400 MHz, CDCl_3_): δ 11.80 (br s, 1H), 7.89 (br s, 1H), 7.82 (s, 1H), 3.78 (t, *J* = 5.6 Hz, 2H), 3.62 (q, *J* = 5.8 Hz, 2H), 1.89–1.83 (m, 2H), 1.74 (s, 9H). ^13^C­{^1^H} NMR (101 MHz, CDCl_3_): δ 161.8, 155.7, 153.7, 140.1, 108.5, 58.6, 57.7, 37.6, 32.4, 29.7. HRMS (ESI, *m*/*z*): [M + H]^+^ calcd for C_12_H_20_N_5_O_2_, 266.1612; found, 266.1610.

## Supplementary Material



## Notes

Copies of ^1^H and ^13^C NMR spectra (PDF)
