# Supplementary material for: Chemical Modification of 2,6,9-Trisubstituted Purine CDK Inhibitors: tert-Butylation at N7/N9 and Access to 2,6,7-Trisubstituted Analogs
Source: ACS Omega. 2026 Apr 14;11(16):23833–44. doi: 10.1021/acsomega.5c10284 (PMC13129827; doi:10.1021/acsomega.5c10284)

Supporting Information

Chemical Modification of 2,6,9-Trisubstituted Purine CDK Inhibitors: *tert*-Butylation at *N*7/*N*9 and Access to 2,6,7-Trisubstituted Analogs

Michal Valenta and Jakub Stýskala*

Department of Organic Chemistry, Faculty of Science, Palacký University, 17. listopadu 12, 771 46 Olomouc, Czech Republic

Email: jakub.styskala@upol.cz

Contents:

^{1}H and ^{13}C NMR data of compounds **2-16** and **22** Pages: S2-S22

NOESY NMR data of compound **4** Pages: S23

7-(*tert*-Butyl)-2,6-dichloro-*7H*-purine (**2**)

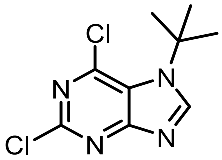

^{1}H NMR (400 MHz, CHLOROFORM-*d*) δ 8.47 (s, 1H), 1.90 (s, 9H)

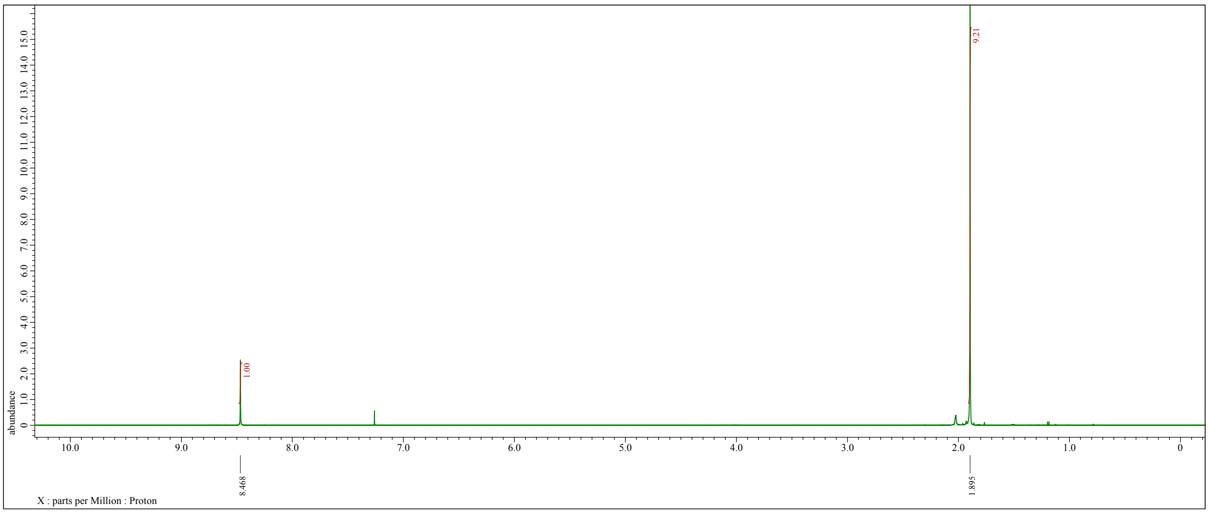

^{13}C{^{1}H} NMR (101 MHz, CHLOROFORM-*d*) δ 165.5, 152.8, 148.3, 143.6, 122.4, 59.6, 31.1

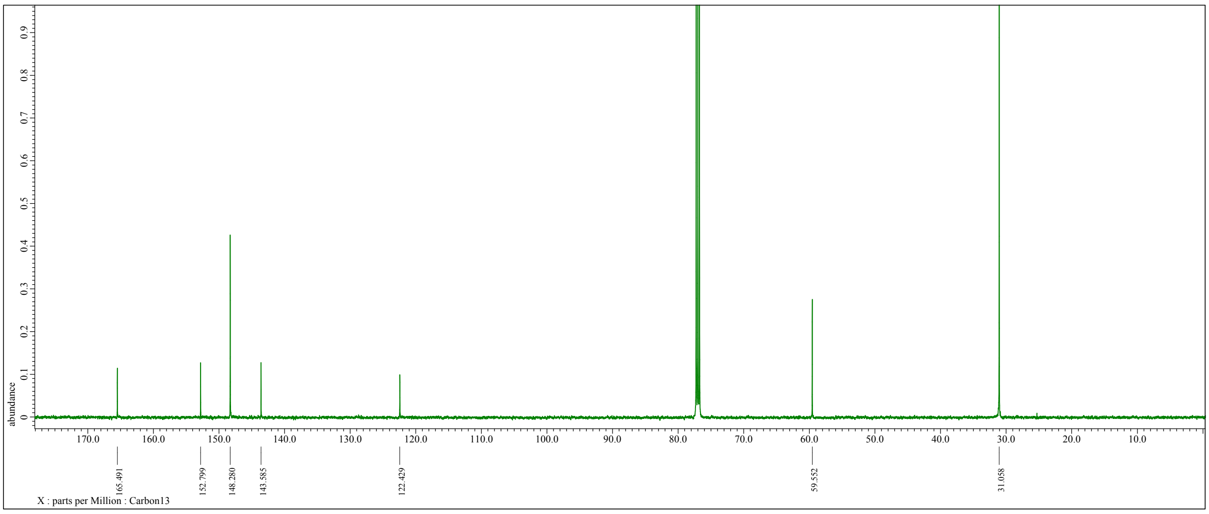

9-(*tert*-Butyl)-2,6-dichloro-9*H*-purine (**3**)

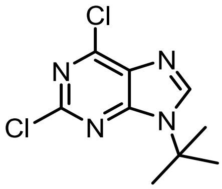

^{1}H NMR (400 MHz, CHLOROFORM-*d*) δ 8.17 (s, 1H), 1.81 (s, 9H)

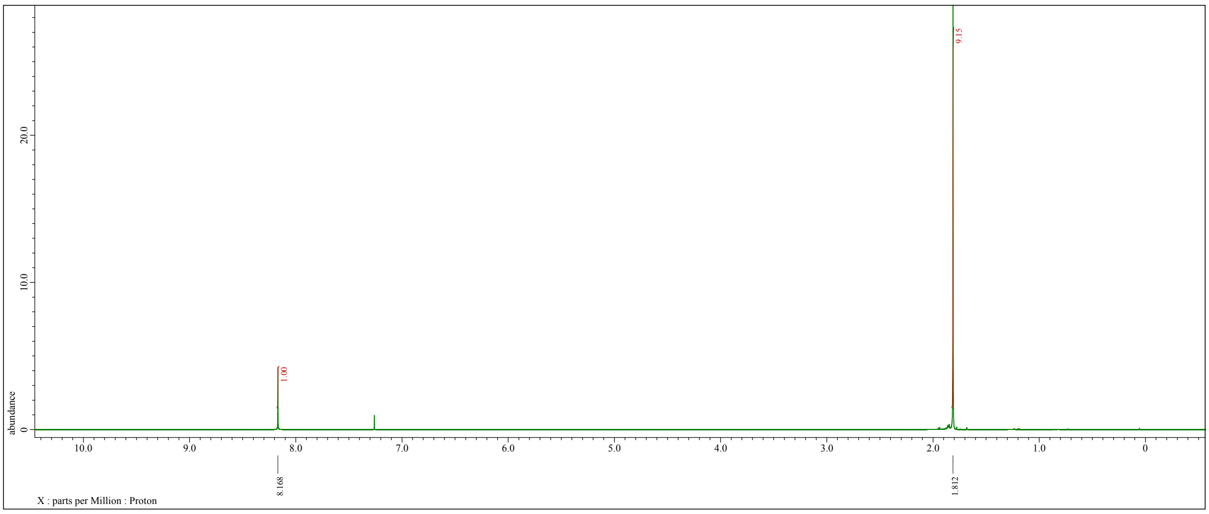

^{13}C{^{1}H} NMR (101 MHz, CHLOROFORM-*d*) δ 153.1, 151.79, 151.76; 143.6, 131.9, 59.0, 28.9

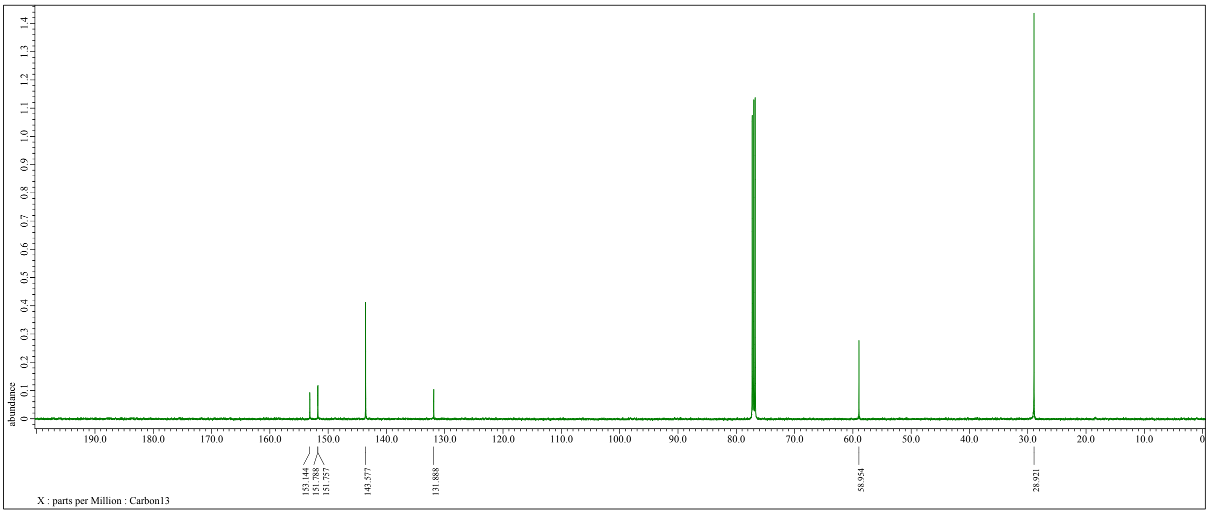

7-(*tert*-Butyl)-2-chloro-6-methoxy-*7H*-purine (**4**)

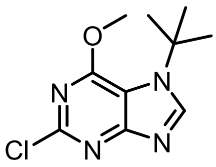

^{1}H NMR (400 MHz, CHLOROFORM-*d*) δ 8.16 (s, 1H), 4.19 (s, 3H), 1.72 (s, 9H)

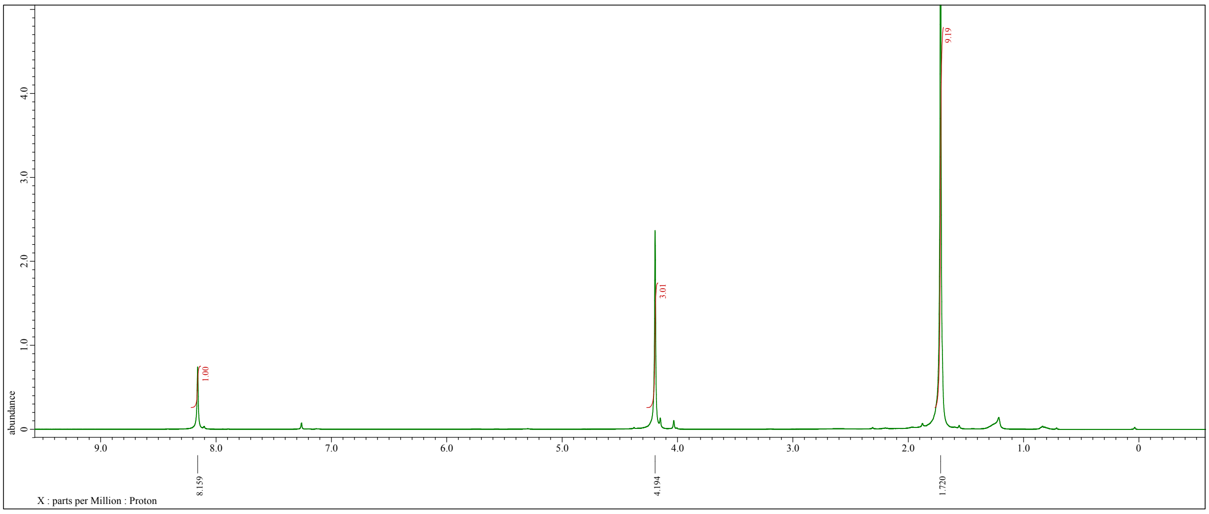

^{13}C{^{1}H} NMR (101 MHz, CHLOROFORM-*d*) δ 164.8, 156.3, 152.5, 144.3, 111.5, 58.3, 55.0, 30.0

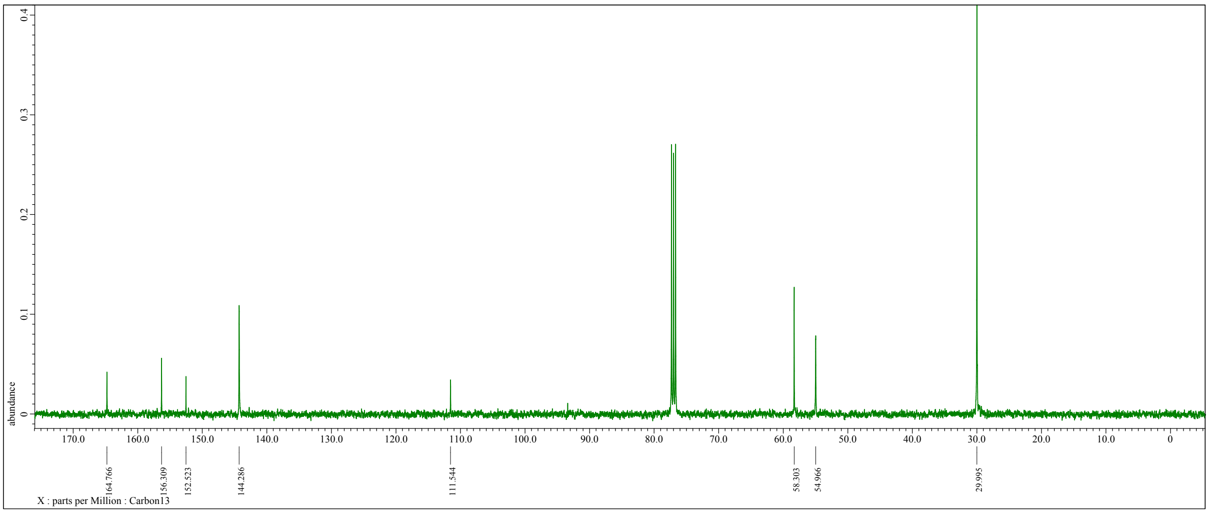

N-Benzyl-9-(*tert*-butyl)-2-chloro-9*H*-purin-6-amine (**5**)

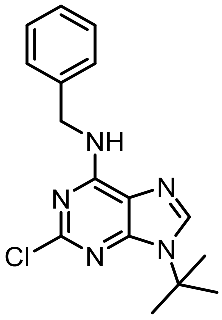

^{1}H NMR (400 MHz, CHLOROFORM-*d*) δ 7.63 (s, 1H), 7.37-7.27 (m, 5H), 6.76 (bs, 1H), 4.83 (bs, 2H), 1.73 (s, 9H)

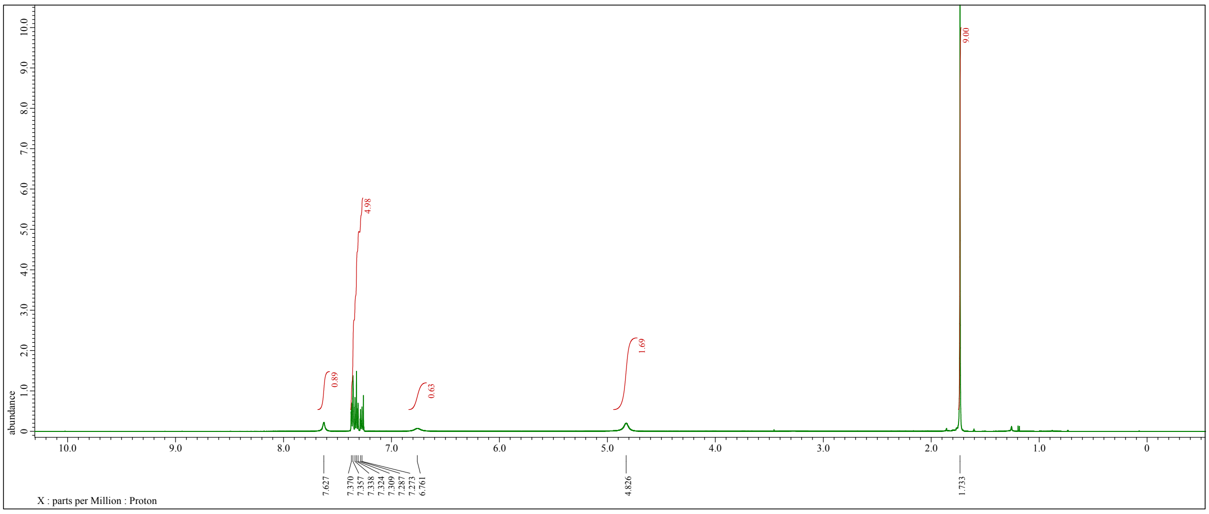

^{13}C{^{1}H} NMR (101 MHz, CHLOROFORM-*d*) δ 155.2, 153.5, 150,4, 138.1, 137.7, 128.7, 127.9, 127.6, 119.7, 57.6, 44.5, 29.0

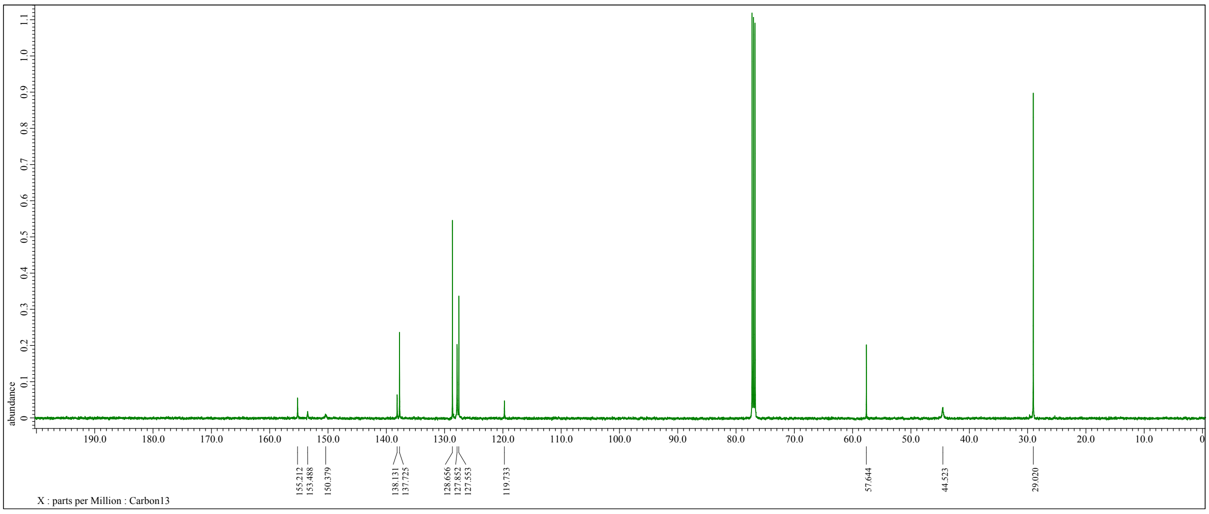

9-(*tert*-Butyl)-2-chloro-*N*-(3-chlophenyl)-9*H*-purin-6-amine (**6**)

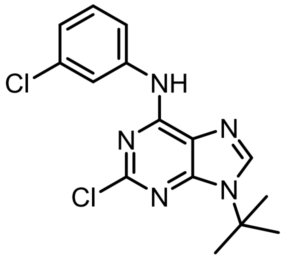

^{1}H NMR (400 MHz, CHLOROFORM-*d*) δ 8.09 (s, 1H), 7.87 (s, 1H), 7.86 (t, *J* = 2.0 Hz, 1H), 7.68 (dd, *J* = 8.1, 2.0 Hz, 1H), 7.29 (t, *J* = 8.1 Hz, 1H), 7.08 (dd, *J* = 7.9, 1.8 Hz, 1H), 1.79 (s, 9H)

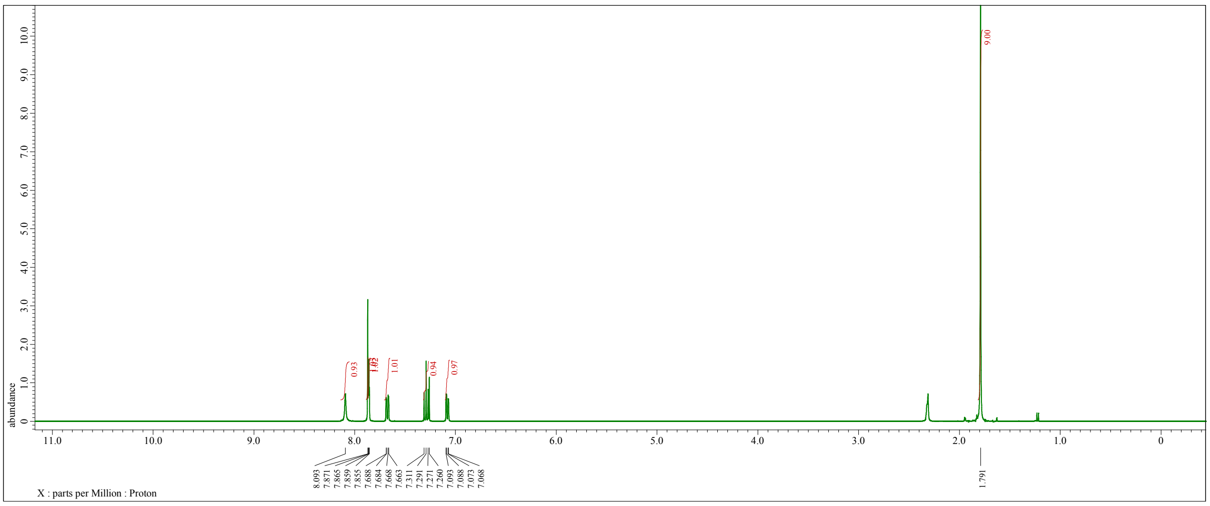

^{13}C{^{1}H} NMR (101 MHz, CHLOROFORM-*d*) δ 152.8, 152.2, 151.0, 139.5, 138.9, 134.6, 130.0, 123.7, 120.6, 120.0, 118.0, 58.0, 29.1

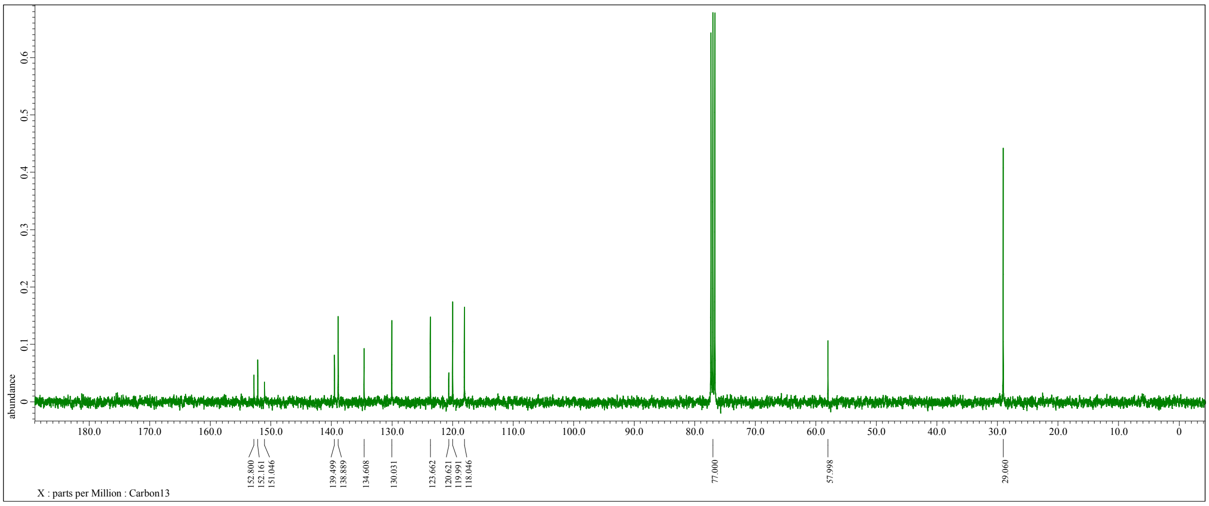

2-((6-(Benzylamino)-9-(*tert*-butyl)-*9H*-purin-2-yl)amino)ethan-1-ol (**7a**)

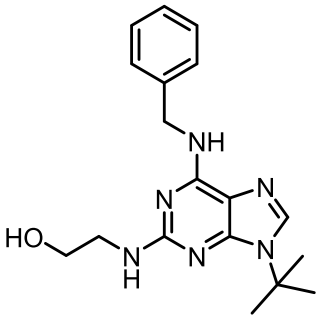

^{1}H NMR (400 MHz, CHLOROFORM-*d*) δ 7.42 (s, 1H), 7.34-7.20 (m, 5H), 6.39 (bs, 1H), 5.27 (t, *J* = 5.8 Hz, 1H), 4.73 (d, *J* = 4.3 Hz, 2H), 4.45-4.67 (bs, 1H), 3.79 (t, *J* = 4.9 Hz, 2H), 3.53 (q, *J* 5.6 Hz, 2H), 1.67 (s, 9H)

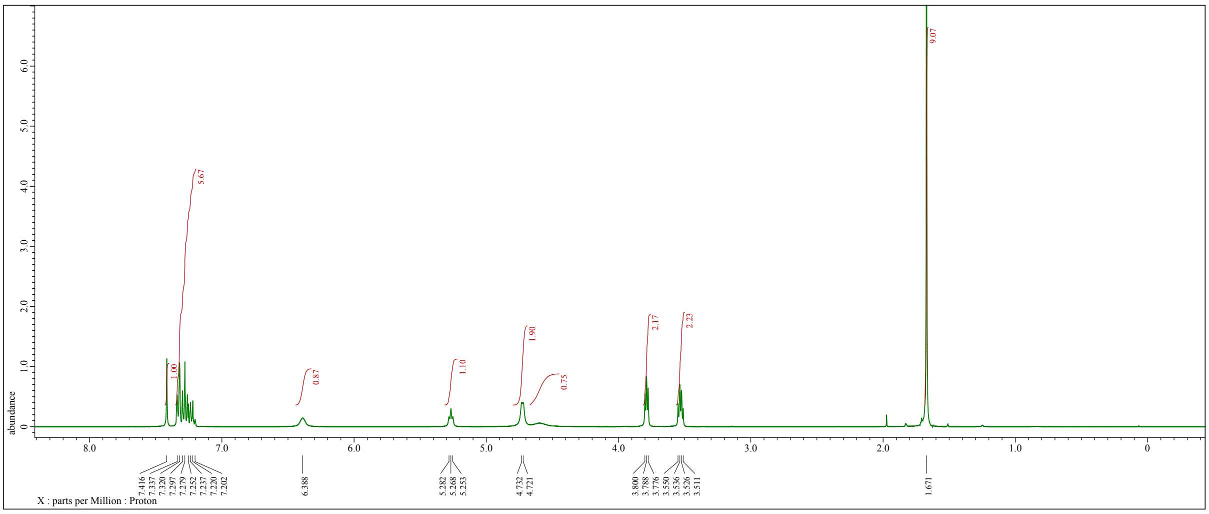

^{13}C{^{1}H} NMR (101 MHz, CHLOROFORM-*d*) δ 159.5, 155.0, 151.0, 139.0, 134.7, 128.5, 127.6, 127.2, 115.8, 64.1, 56.4, 45.0, 44.3, 28.9

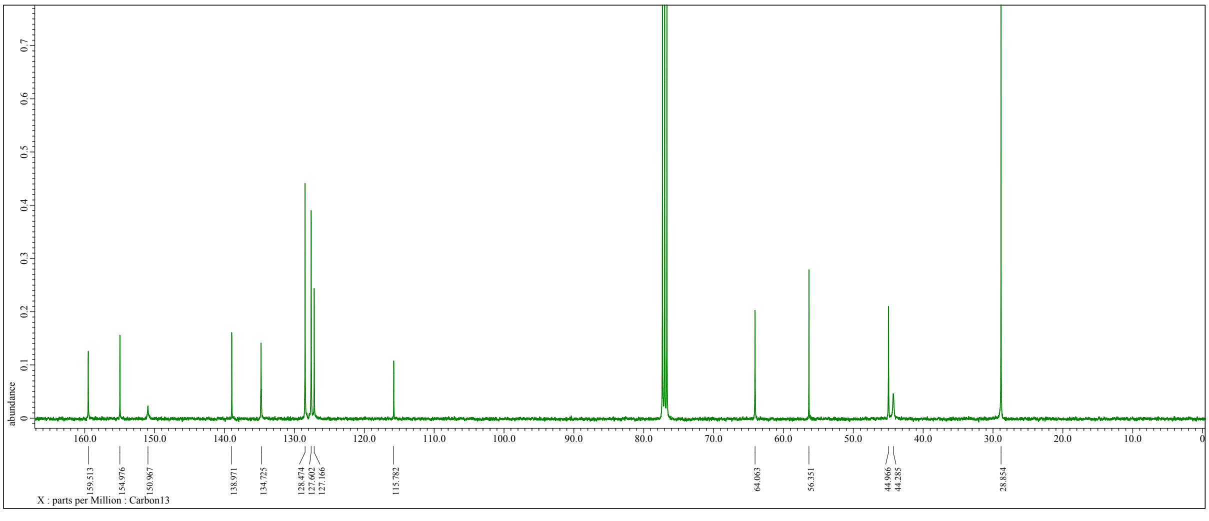

3-((6-(Benzylamino)-9-(*tert*-butyl)-*9H*-purin-2-yl)amino)propan-1-ol (**7b**)

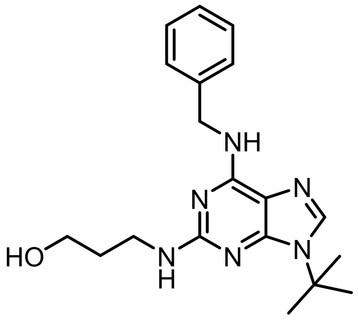

^{1}H NMR (400 MHz, CHLOROFORM-*d*) δ 7.46 (s, 1H), 7.36-7.22 (m, 5H), 6.19 (bs, 1H), 4.95 (t, *J* = 6.3 Hz, 1H), 4.75 (d, *J* = 4.3 Hz, 2H), 3.70 (t, *J* = 5.8 Hz, 2H), 3.58 (q, *J* = 6.3 Hz, 2H), 1.78 (quin, *J* = 6.2 Hz, 2H), 1.70 (s, 9H)

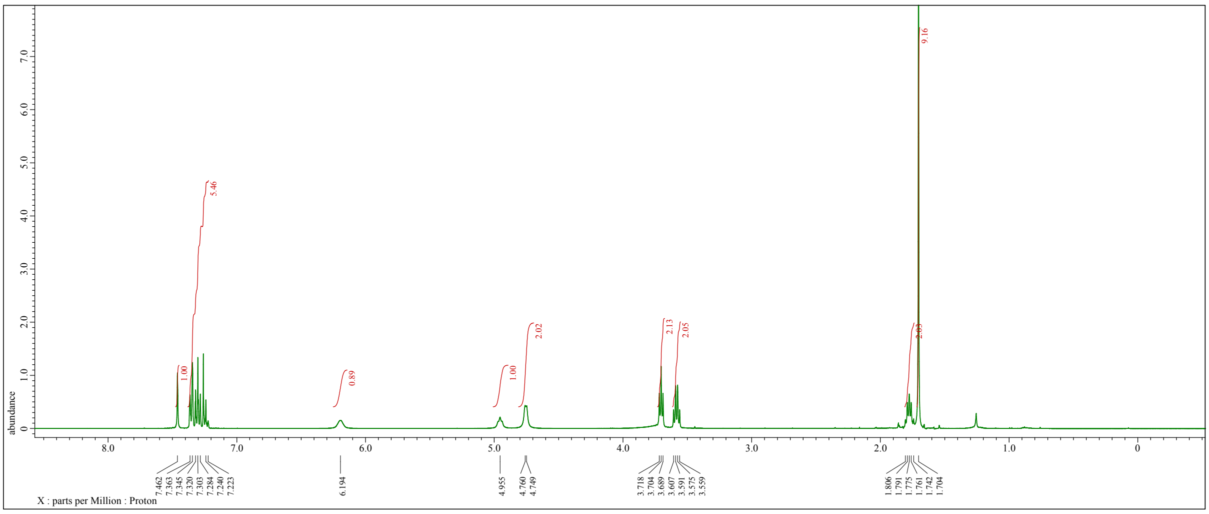

^{13}C{^{1}H} NMR (101 MHz, CHLOROFORM-*d*) δ 159.3, 155.0, 151.4, 138.9, 134.7, 128.5, 127.7, 127.2, 115.5, 59.3, 56.4, 44.4, 38.0, 33.0, 29.0

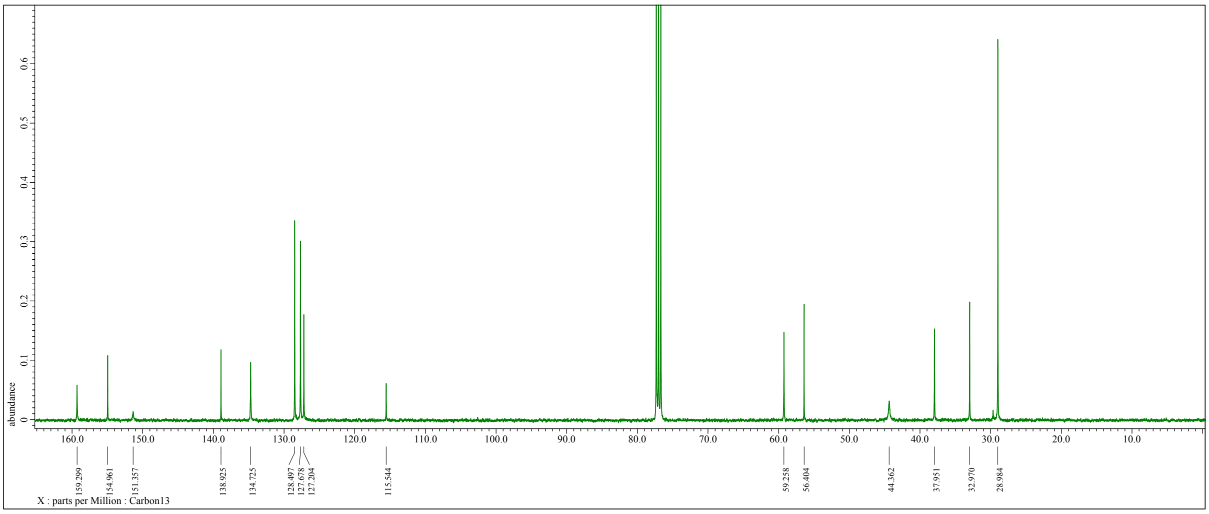

(*R*)-2-((6-(Benzylamino)-9-(*tert*-butyl)-9*H*-purin-2-yl)amino)butan-1-ol (**7c**)

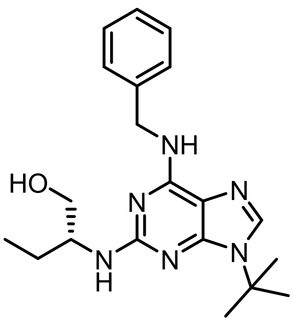

^{1}H NMR (400 MHz, CHLOROFORM-*d*) δ 7.45 (s, 1H), 7.36-7.23 (m, 5H), 6.18 (bs, 1H), 4.87 (d, *J* = 6.2 Hz, 1H), 4.78-4.68 (m, 2H), 4.44 (bs, 1H), 3.89 (quind, *J* = 7.0, 3.1 Hz, 1H), 3.82 (dd, *J* = 10.7, 3.1 Hz, 1H), 3.62 (dd, *J* = 10.7, 7.6 Hz, 1H), 1.69 (s, 9H), 1.58 (decaplet, *J* = 7.3 Hz, 2H), 1.00 (t, *J* = 7.5 Hz, 3H)

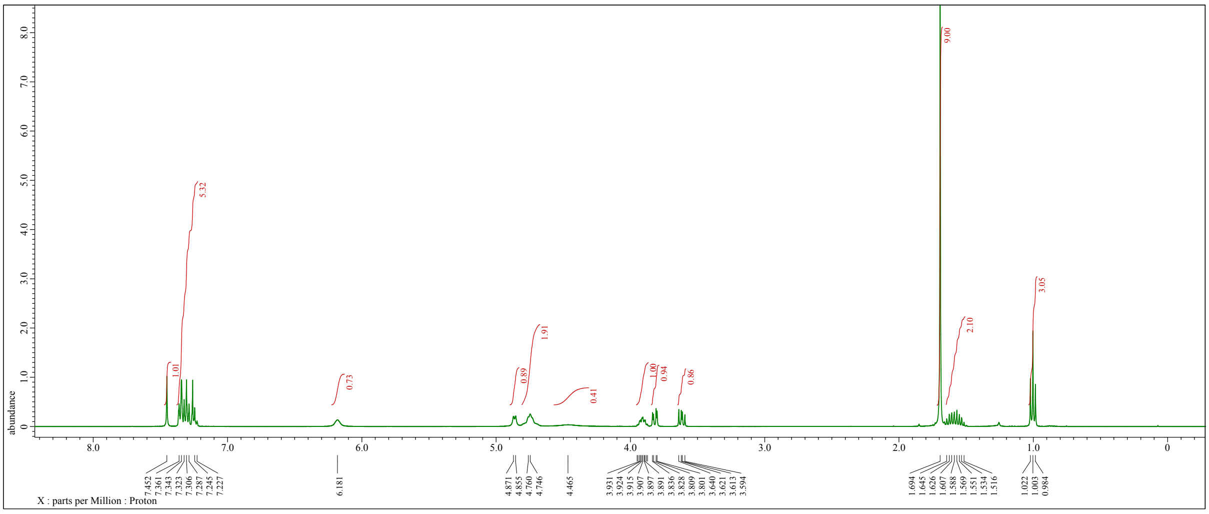

^{13}C{^{1}H} NMR (101 MHz, CHLOROFORM-*d*) δ 159.3, 154.9, 150.9, 138.9, 134.8, 128.5, 127.7, 127.3, 115.8, 67.7, 56.4, 55.9, 44.4, 28.9, 24.9, 10.9

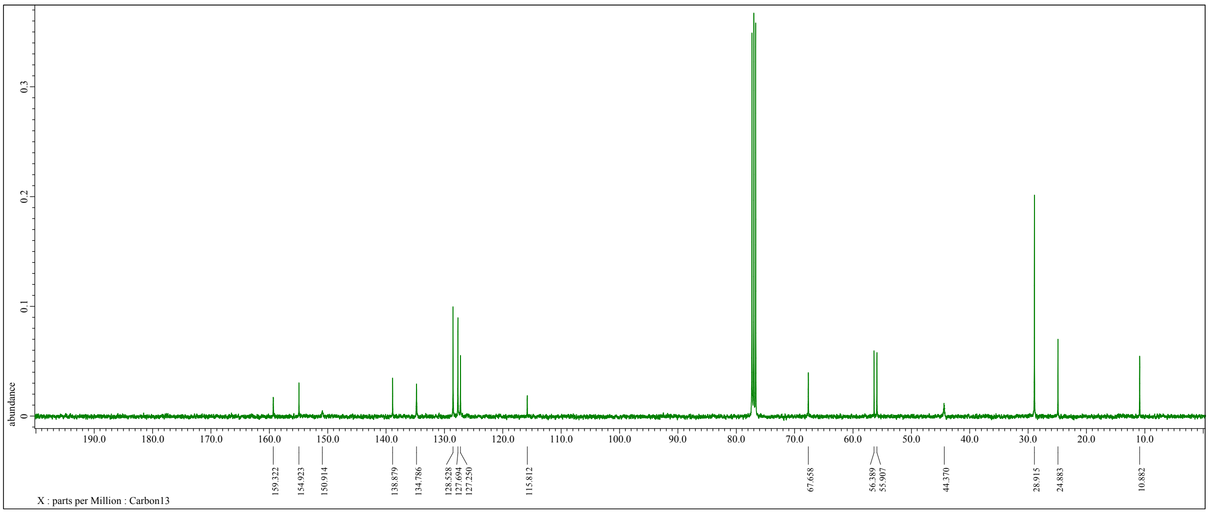

(*R*)-2-((9-(*tert*-Butyl)-6-((3-chlorophenyl)amino)-9*H*-purin-2-yl)amino-3-methylbutan-1-ol (**8**)

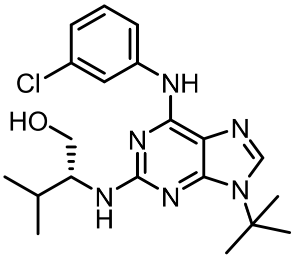

^{1}H NMR (400 MHz, CHLOROFORM-*d*) δ 7.97 (s, 1H), 7.90 (s, 1H), 7.57 (s, 1H), 7.46 (d, *J* = 8.2 Hz, 1H), 7.19 (t, *J* = 8.1 Hz, 1H), 6.98 (dd, *J* = 7.9, 0.9 Hz, 1H), 5.04 (d, *J* = 7.9 Hz, 1H), 4.00-3.94 (m, 1H), 3.91 (d, *J* = 10.4 Hz, 1H), 3.81 (bs, 1H), 3.75 (dd, *J* = 10.2, 7.5 Hz, 1H), 2.02 (td, *J* = 13.2, 7.0 Hz, 1H), 1.69 (s, 9H), 1.04 (d, *J* = 6.7 Hz, 6H)

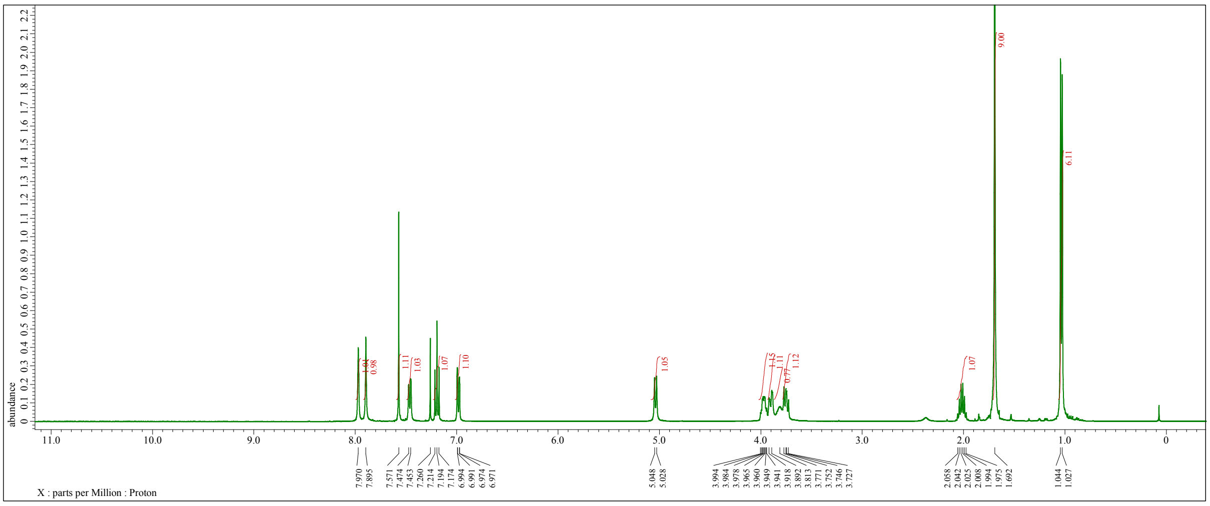

^{13}C{^{1}H} NMR (101 MHz, CHLOROFORM-*d*) δ 159.1, 152.1, 151.6, 140.6, 135.5, 134.3, 129.7, 122.5, 119.7, 117.6, 116.1, 64.6, 59.3, 56.6, 29.9, 28.9, 19.5, 19.1

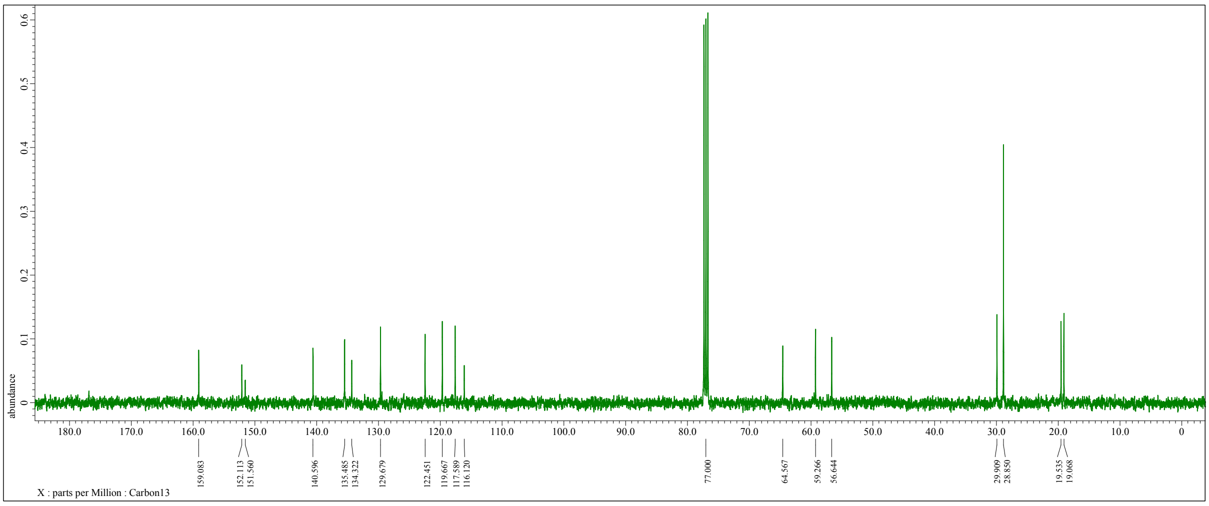

N-Benzyl-7-(*tert*-butyl)-2-chloro-7*H*-purin-6-amine (**9**)

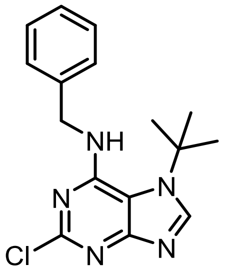

^{1}H NMR (400 MHz, CHLOROFORM-*d*) δ 8.10 (s, 1H), 7.39-7.31 (m, 5H), 5.49 (bs, 1H), 4.85 (d, *J* = 5.0 Hz, 2H), 1.73 (s, 9H)

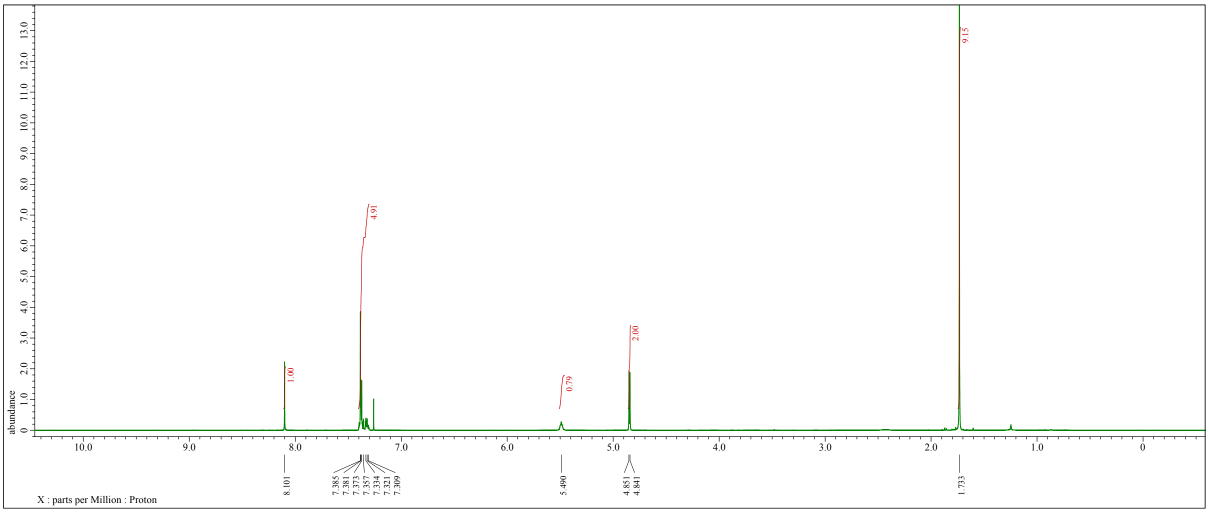

^{13}C{^{1}H} NMR (101 MHz, CHLOROFORM-*d*) δ 163.1, 154.1, 150.2, 142.9, 137.6, 129.0, 127.9 (2 x C), 110.7, 56.5, 46.3, 31.6

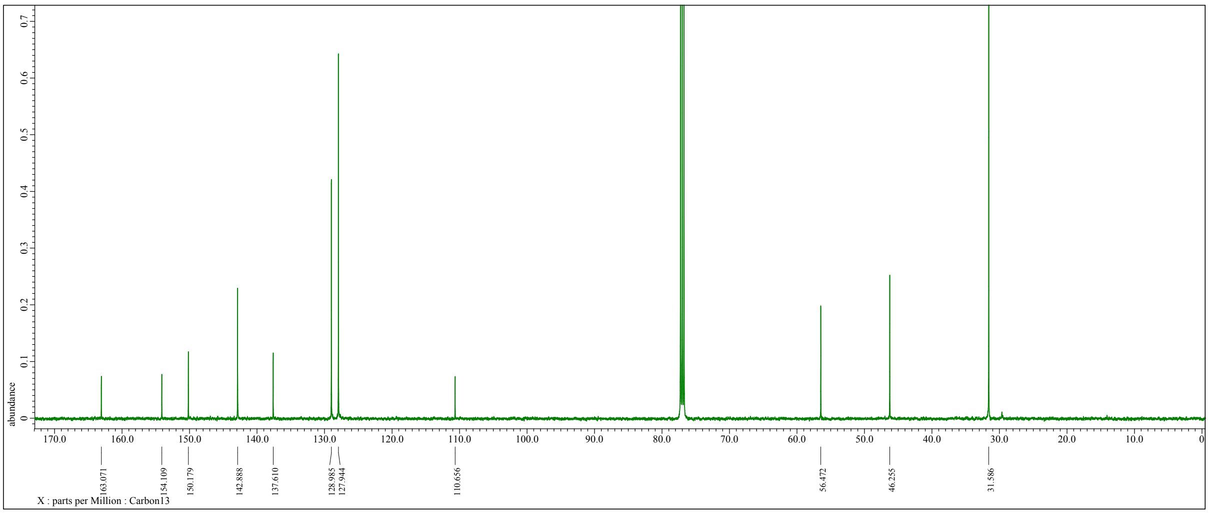

7-(*tert*-Butyl)-2-chloro-*N*-(3-chlorophenyl)-7*H*-purin-6-amine (**10**)

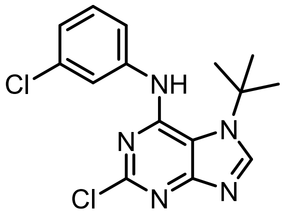

^{1}H NMR (400 MHz, DMSO-d_6) δ 8.59 (s, 1H), 7.99 (s, 1H), 7.62 (s, 1H), 7.47-7.39 (m, 2H), 7.24-7.22 (m, 1H), 1.78 (s, 9H)

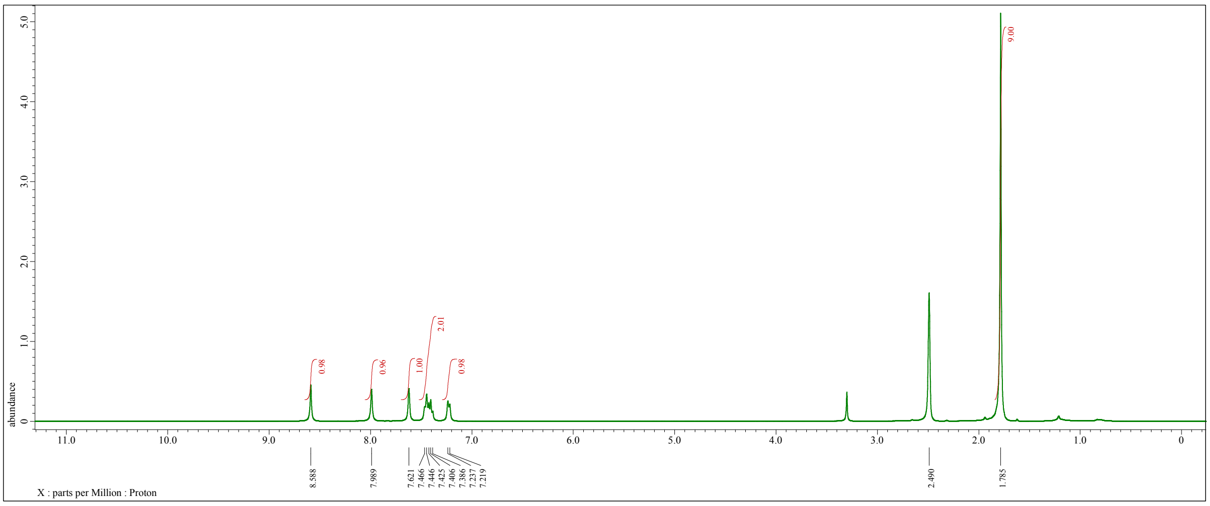

^{13}C{^{1}H} NMR (101 MHz, DMSO-d_6) δ 163.8, 151.6, 148.6, 146.3, 140.2, 132.7, 130.0, 124.2, 123.4, 122.4, 111.8, 57.6, 31.0

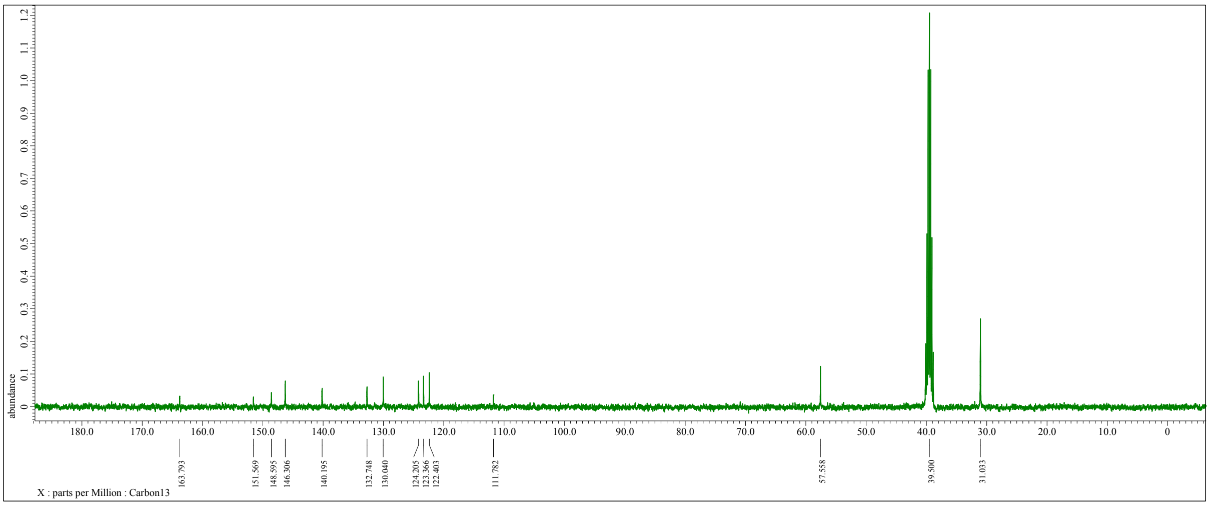

2-((6-(Benzylamino)-7-(*tert*-butyl)-7*H*-purin-2-yl)amino)ethan-1-ol (**11a**)

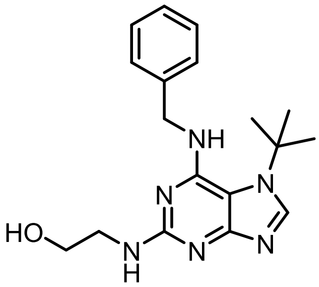

^{1}H NMR (400 MHz, DMSO-d_6) δ 8.01 (s, 1H), 7.37-7.17 (m, 5H), 6.39 (t, J = 5.2 Hz, 1H), 5.90 (t, J = 5.8 Hz, 1H), 4.76 (d, J = 5.5 Hz, 2H), 3.44 (t, J = 6.0 Hz, 2H), 3.25 (q, J = 5.9 Hz, 2H), 1.68 (s, 9H). Trace amount of ammonium acetate detected at 1.90 ppm.

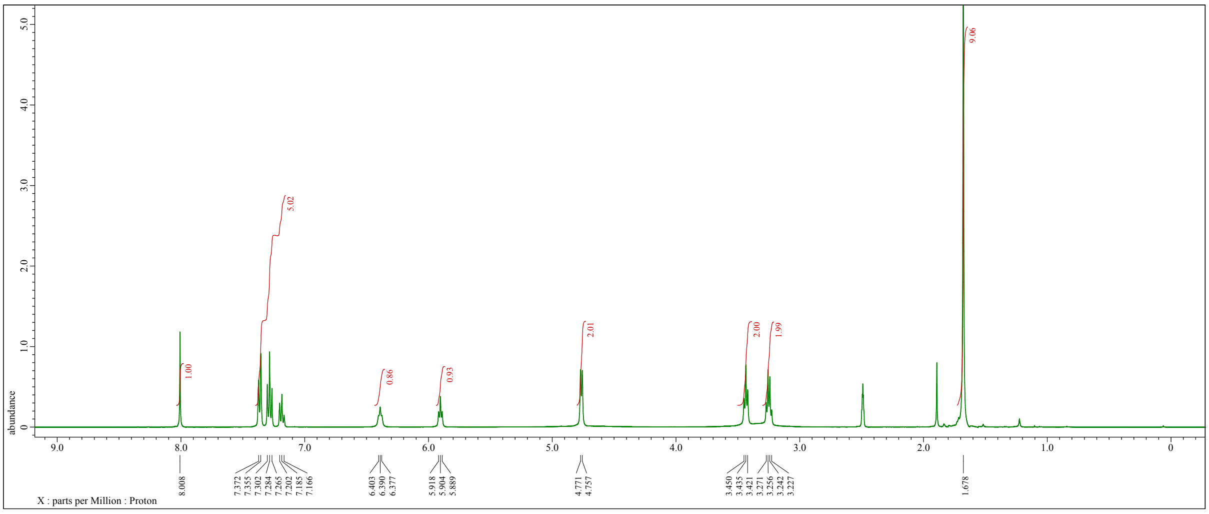

^{13}C{^{1}H} NMR (101 MHz, DMSO-d_6) δ 163.4, 158.5, 149.4, 141.7, 140.6, 128.1, 127.1, 126.3, 105.5, 60.6, 55.6, 44.1, 43.8, 30.9

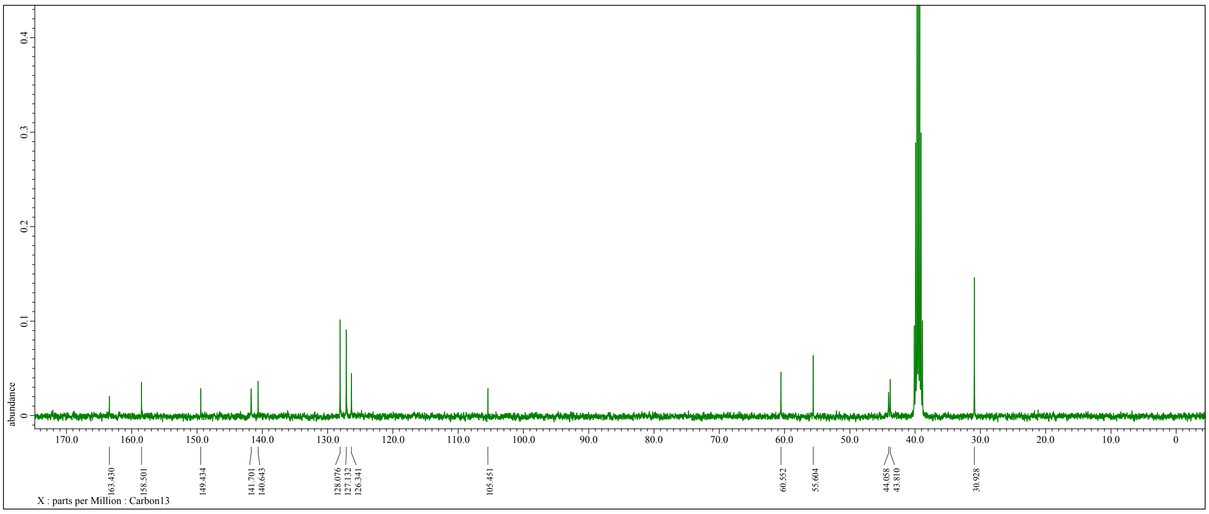

3-((6-(Benzylamino)-7-(*tert*-butyl)-*7H*-purin-2-yl)amino)propan-1-ol (**11b**)

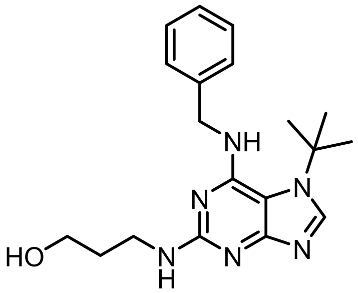

^{1}H NMR (400 MHz, DMSO-d_6) δ 8.00 (s, 1H), 7.38-7.16 (m, 5H), 6.38 (bs, 1H), 6.06 (t, *J* = 6.2 Hz, 1H), 4.78 (d, *J* = 4.9 Hz, 2H), 3.41 (t, *J* = 6.3 Hz, 2H), 3.23 (q, *J* = 6.4 Hz, 2H), 1.68 (s, 9H), 1.58 (quin, *J* = 6.4 Hz, 2H). Trace amount of ammonium acetate detected at 1.90 ppm.

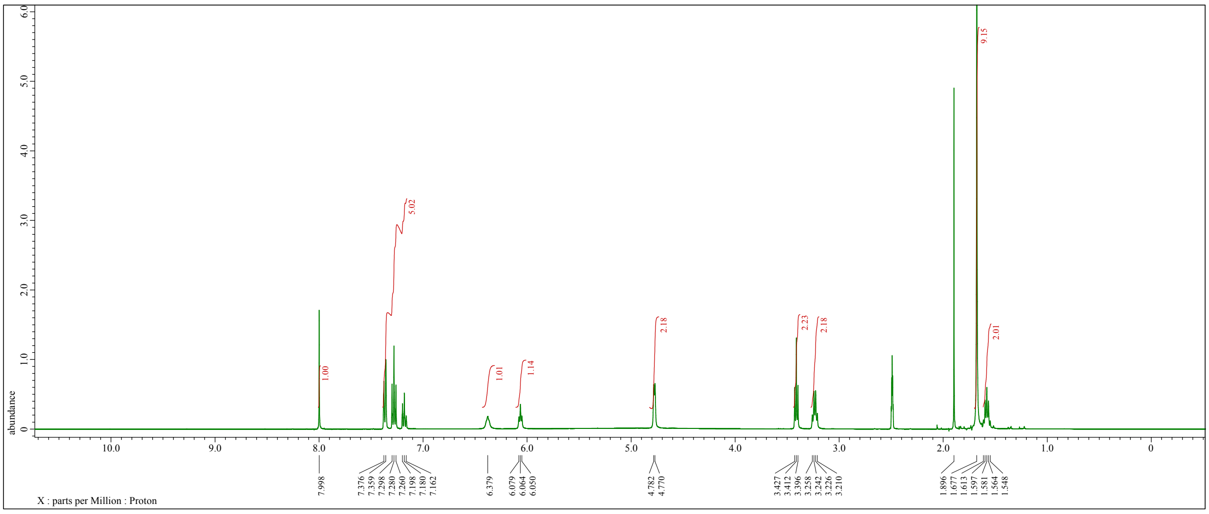

^{13}C{^{1}H} NMR (101 MHz, DMSO-d_6) δ 163.5, 158.6, 149.4, 141.6, 140.7, 128.1, 127.1, 126.3, 105.3, 58.7, 55.6, 44.1, 38.1, 32.8, 30.9

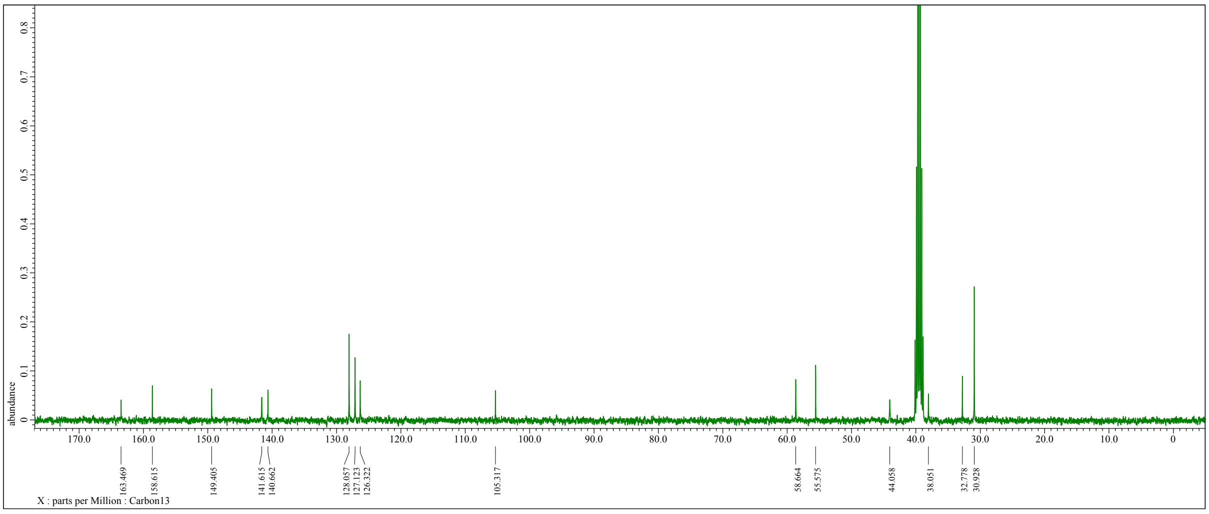

(*R*)-2-((6-(Benzylamino)-7-(*tert*-butyl)-7*H*-purin-2-yl)amino)butan-1-ol (**11c**)

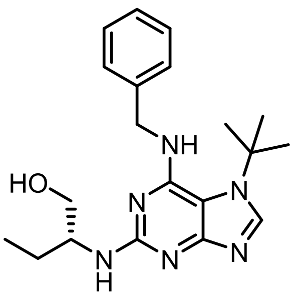

^{1}H NMR (400 MHz, DMSO-d_6) δ 8.00 (s, 1H), 7.37-7.16 (m, 5H), 6.36 (bs, 1H), 5.59 (d, *J* = 8.2 Hz, 1H), 4.77 (ddd, *J* = 24.9, 15.0, 5.3 Hz, 2H), 3.72 (td, *J* = 13.3, 5.4 Hz, 1H), 3.42 (dd, *J* = 10.5, 4.7 Hz, 1H), 3.29 (dd, *J* = 10.5, 5.6 Hz, 1H), 1.68 (s, 9H), 1.59-1.52 (m, 1H), 1.43-1.32 (m, 1H), 0.78 (t, *J* = 7.3 Hz, 3H). Trace amount of ammonium acetate detected at 1.90 ppm.

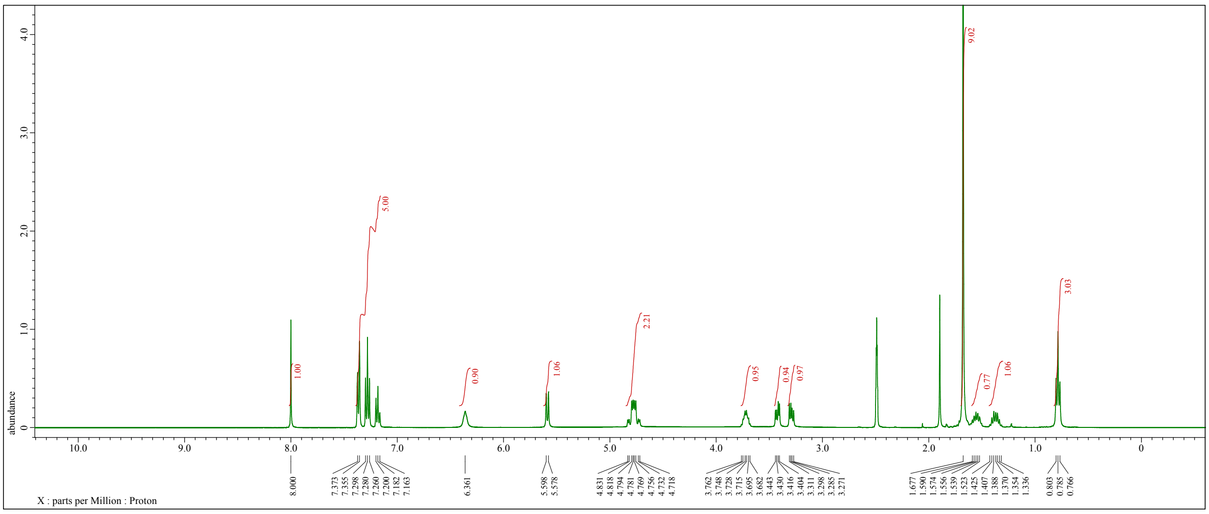

^{13}C{^{1}H} NMR (101 MHz, DMSO-d_6) δ 163.2, 158.4, 149.4, 141.7, 140.6, 128.1, 127.1, 126.3, 105.3, 63.2, 55.7, 53.8, 44.1, 30.9, 23.8, 10.5

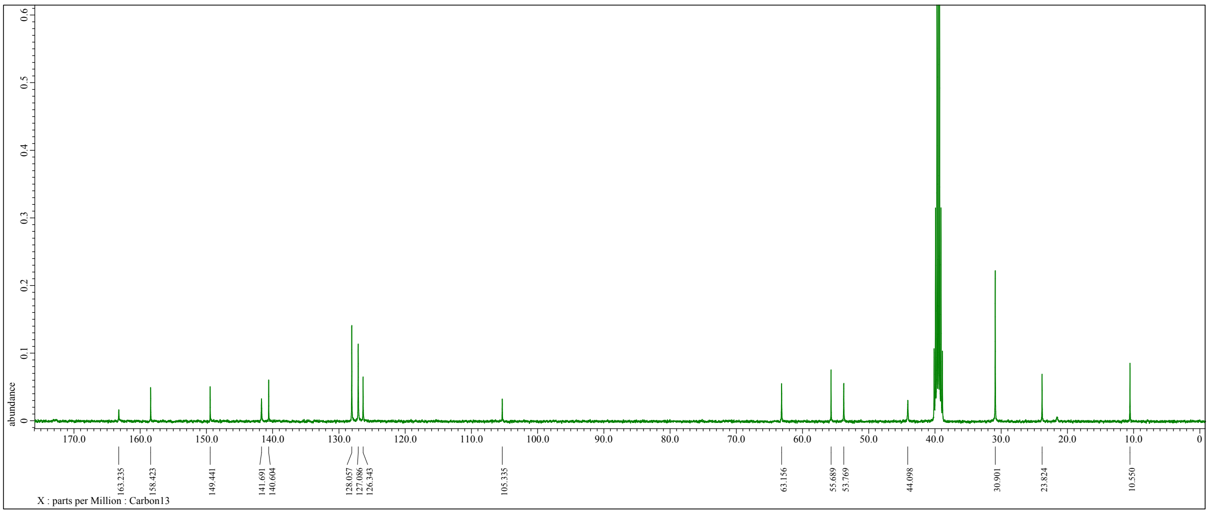

2-((4-Amino-6-(benzylamino)-5-(*tert*-butylamino)pyrimidin-2-yl)amino)ethan-1-ol (**12a**)

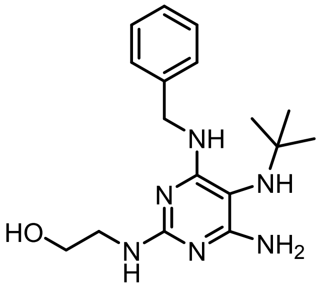

^{1}H NMR (400 MHz, CHLOROFORM-*d*) δ 7.32-7.25 (m, 5H), 5.94 (t, *J* = 5.2 Hz, 1H), 5.51 (bs, 1H), 4.55 (d, *J* = 5.8 Hz, 2H), 3.74 (t, *J* = 4.7 Hz, 2H), 3.47 (q, *J* = 4.6 Hz, 2H), 1.13 (s, 9H). Trace amount of ammonium acetate detected at 1.95 ppm.

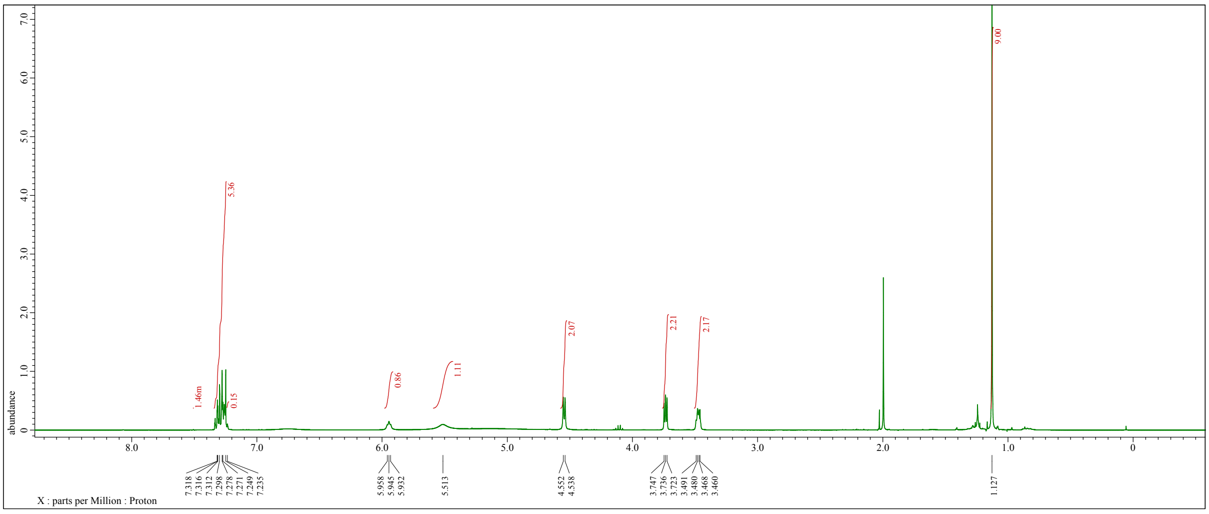

^{13}C{^{1}H} NMR (101 MHz, CHLOROFORM-*d*) δ 162.7, 154.4, 153.1, 138.5, 128.6, 127.5, 127.4, 92.7, 61.8, 54.6, 45.0, 44.1, 30.6. Trace amount of ammonium acetate detected at 23 and 178 ppm.

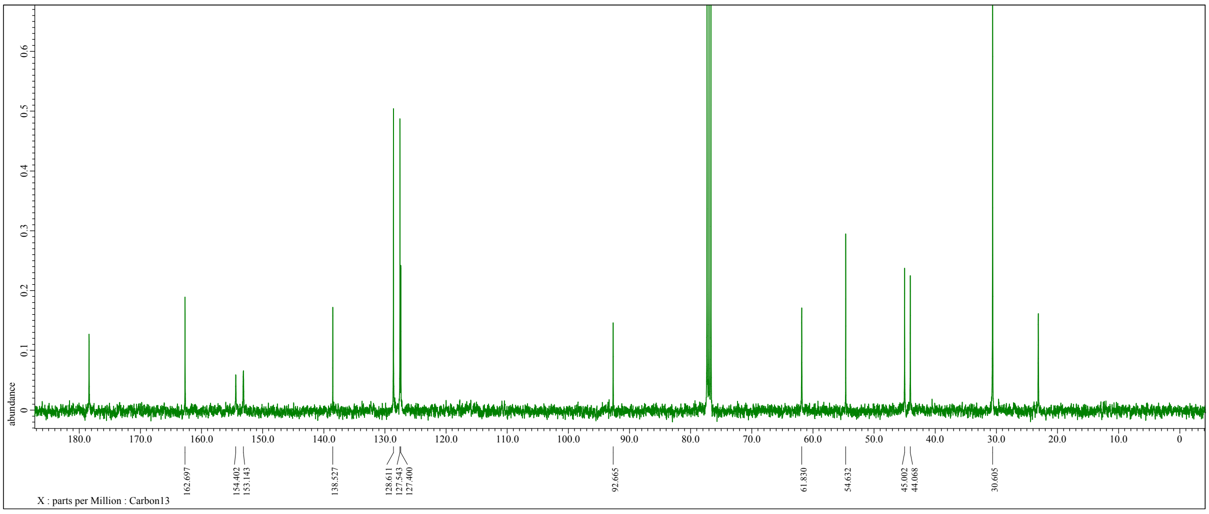

3-((4-Amino-6-(benzylamino)-5-(*tert*-butylamino)pyrimidin-2-yl)amino)propan-1-ol (**12b**)

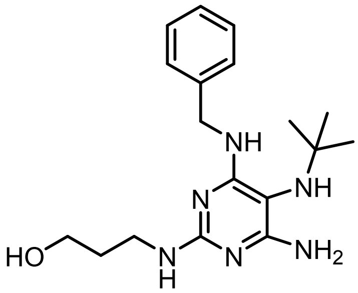

^{1}H NMR (400 MHz, CHLOROFORM-*d*) δ 7.33-7.25 (m, 5H), 5.72 (bs, 1H), 4.94 (bs, 1H), 4.53 (d, *J* = 5.8 Hz, 2H), 3.62 (t, *J* = 5.6 Hz, 2H), 3.49 (q, *J* = 6.1 Hz, 2H), 1.67 (quin, *J* = 5.8 Hz, 2H), 1.13 (s, 9H). Trace amount of ammonium acetate detected at 1.95 ppm.

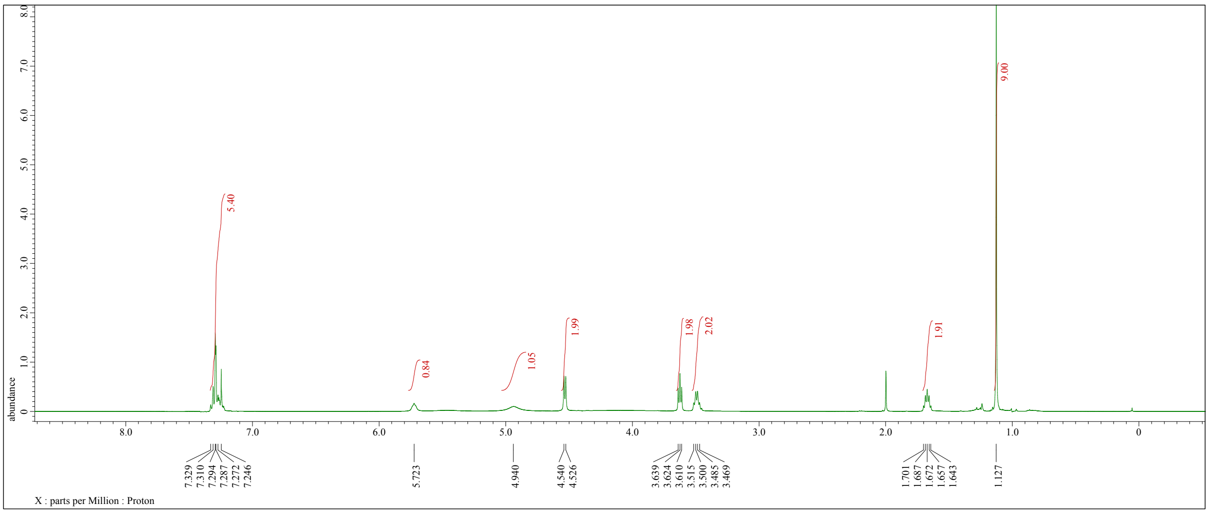

^{13}C{^{1}H} NMR (101 MHz, CHLOROFORM-*d*) δ 162.4, 160.6, 159.2, 139.7, 128.5, 127.6, 127.1, 94.8, 58.1, 54.3, 45.0, 37.0, 33.6, 30.9

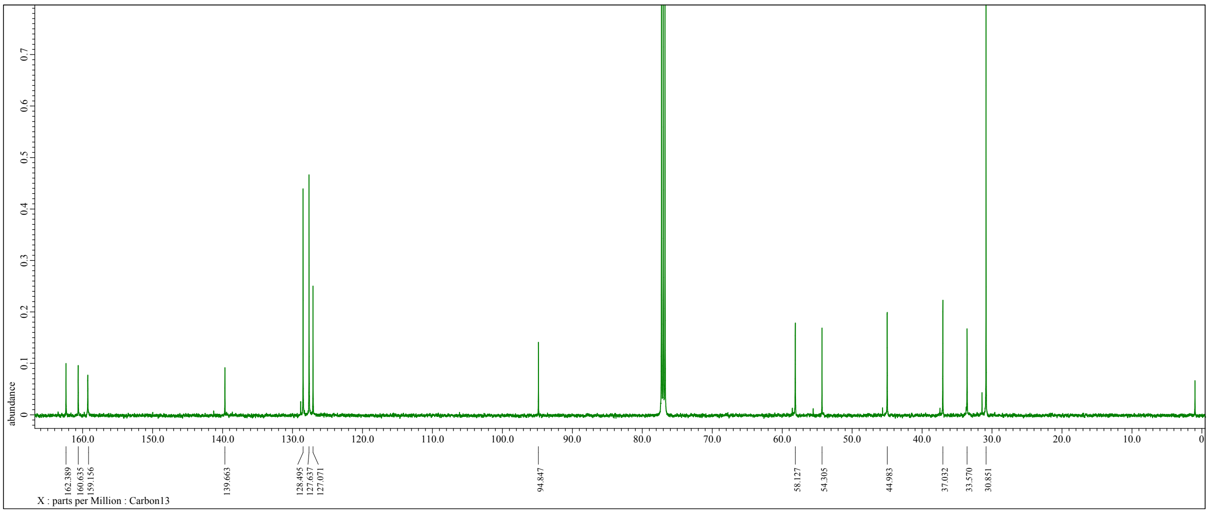

(*R*)-2-((7-(*terc*-Butyl)-6-((3-chlorophenyl)amino)-7*H*-purin-2-yl)amino-3-methylbutan-1-ol (**13**)

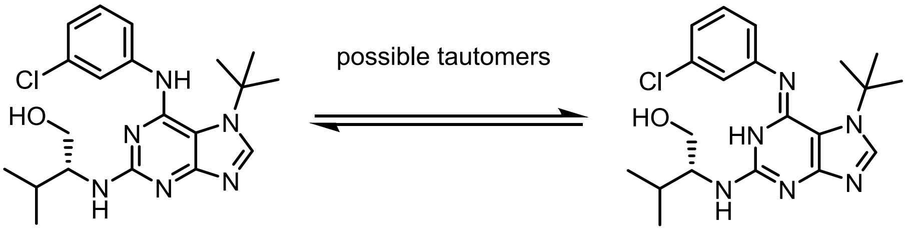

^{1}H NMR (400 MHz, DMSO-d_6) δ 9.19 (s, 1H), 8.19 (s, 1H), 7.86 (s, 1H), 7.64 (s, 1H), 7.46 (s, 2H), 7.32 (dd, J = 18.2, 8.1 Hz, 2H), 7.04 (t, *J* = 6.6 Hz, 2H), 6.86 (s, 1H), 6.80 (d, *J* = 7.9 Hz, 1H), 6.54 (d, *J* = 8.5 Hz, 1H), 5.95 (d, *J* = 8.9 Hz, 1H), 4.67 (s, 1H), 4.46 (s, 1H), 3.72-3.65 (m, 2H), 3.47-3.37 (m, 4H), 1.87-1.80 (m, 2H), 1.73 (s, 18H), 0.88-0.83 (m, 12H). Trace amount of ammonium acetate detected at 1.90 ppm.

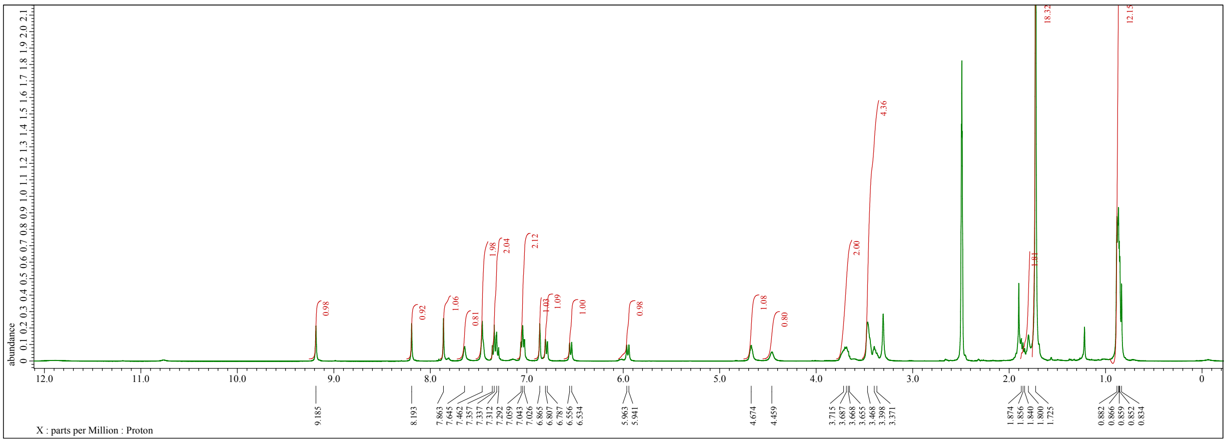

^{13}C{^{1}H} NMR (101 MHz, DMSO-d_6) δ 165.0, 158.7, 158.2, 151.1, 150.3, 147.1, 144.3, 141.9, 140.2, 139.6, 133.8, 132.8, 131.1, 130.0, 121.8, 121.6, 121.5, 120.6, 120.2, 119.4, 108.2, 107.3, 61.3, 60.7, 57.6, 57.4, 56.6, 56.3, 30.9, 29.6, 28.6, 28.1, 19.4, 19.3, 18.8, 18.4. Trace amount of ammonium acetate detected at 21 and 172 ppm.

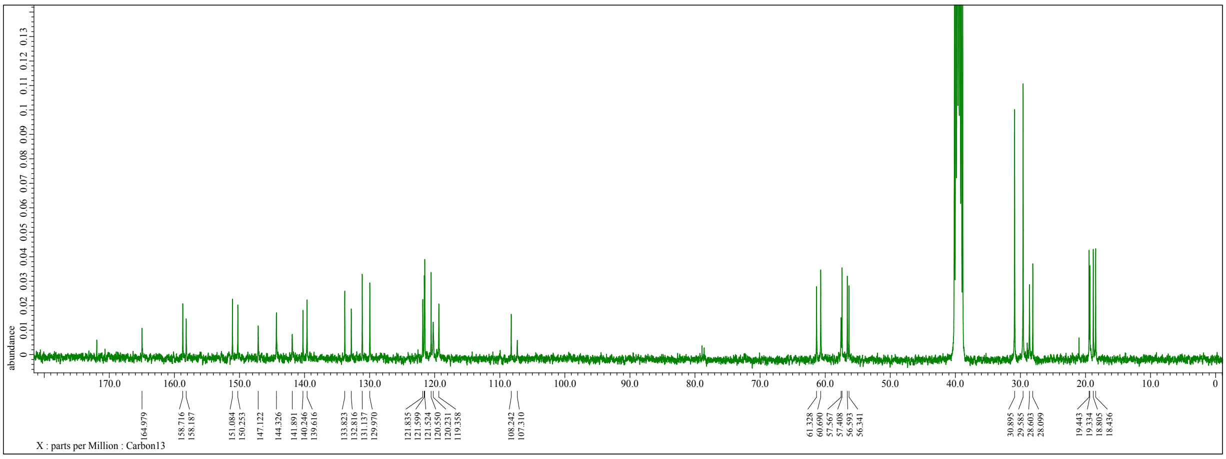

(*R*)-2-((4-Amino-5-(*tert*-butylamino)-6-((3-chlorophenyl)amino)pyrimidin-2-yl)amino)-3-methylbutan-1-ol (**14**)

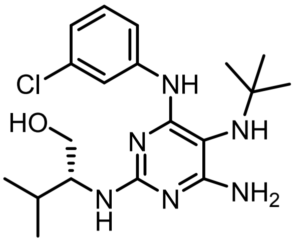

^{1}H NMR (400 MHz, CHLOROFORM-*d*) δ 7.99 (bs, 1H), 7.84 (s, 1H), 7.34-7.28 (m, 1H), 7.15 (t, *J* = 7.9 Hz, 1H), 6.93 (d, *J* = 7.9 Hz, 1H), 5.23 (bs, 2H), 3.84-3.81 (m, 2H), 3.71-3.67 (m, 1H), 1.98-1.92 (m, 1H), 1.18 (s, 9H), 1.0,0 (s, 6H).

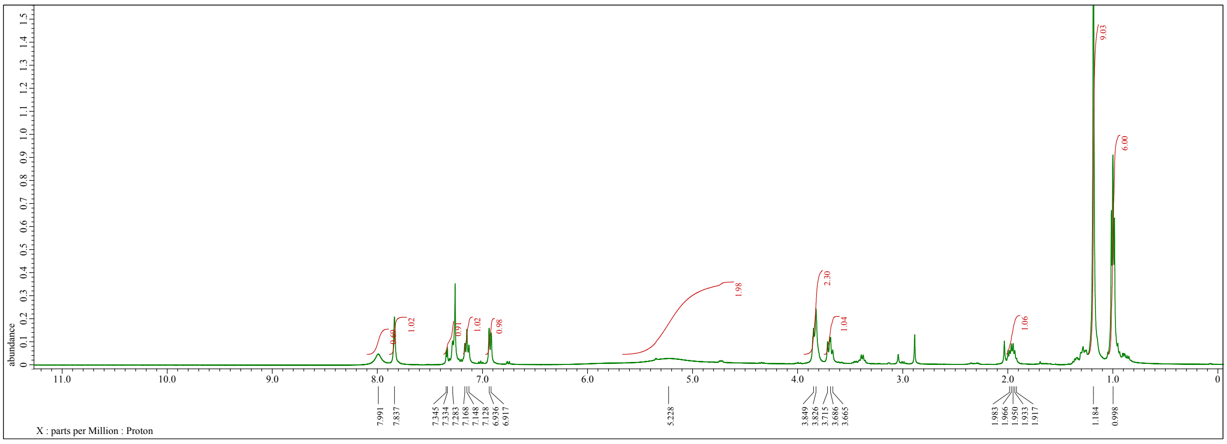

^{13}C{^{1}H} NMR (101 MHz, CHLOROFORM-*d*) δ 160.8, 159.7, 158.8, 141.3, 134.4, 129.6, 121.4, 118.8, 116.7, 96.0, 59.1, 54.4, 30.8, 29.9, 29.7, 19.2, 19.1

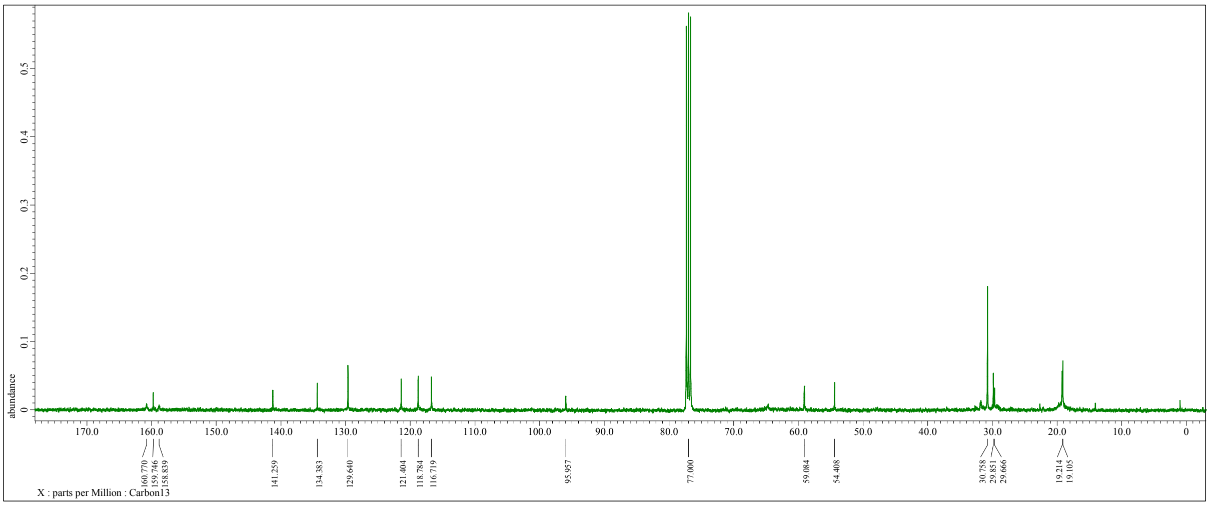

N[6]-Benzyl-7-(*tert*-butyl)-*N*[2], *N*[2]-dimethyl-*7H*-purin-2,6-diamine (**15**)

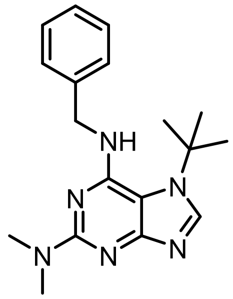

^{1}H NMR (400 MHz, CHLOROFORM-*d*) δ 7.87 (s, 1H), 7.38-7.28 (m, 5H), 5.15 (t, *J* = 5.3 Hz, 1H), 4.83 (d, *J* = 5.3 Hz, 2H), 3.16 (s, 6H), 1.69 (s, 9H)

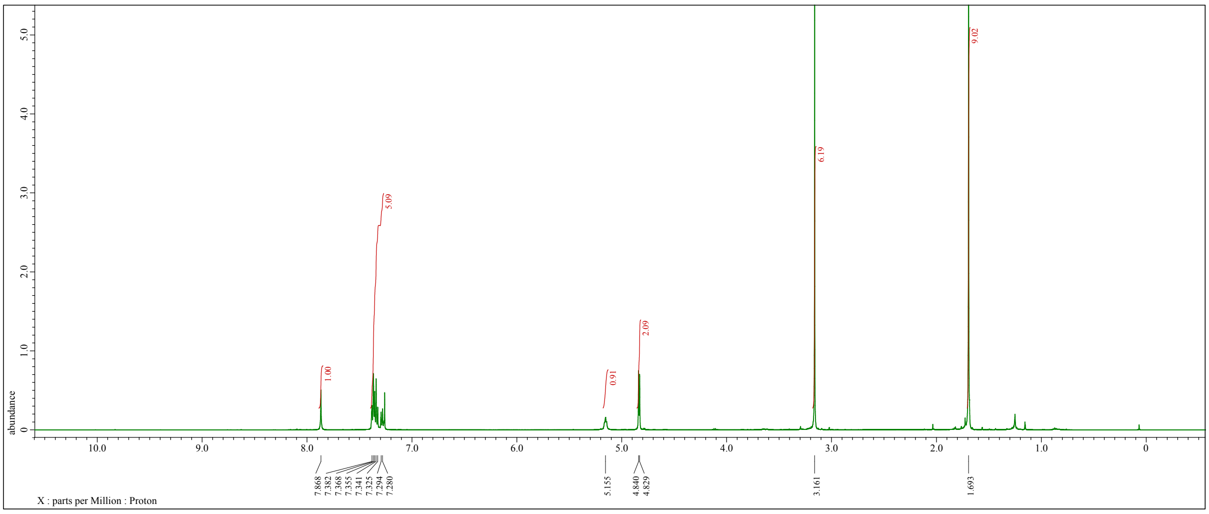

^{13}C{^{1}H} NMR (101 MHz, CHLOROFORM-*d*) δ 164.5, 159.6, 149.3, 141.0, 139.2, 128.7, 127.5, 127.3, 105.2, 55.3, 45.6, 37.4, 31.4

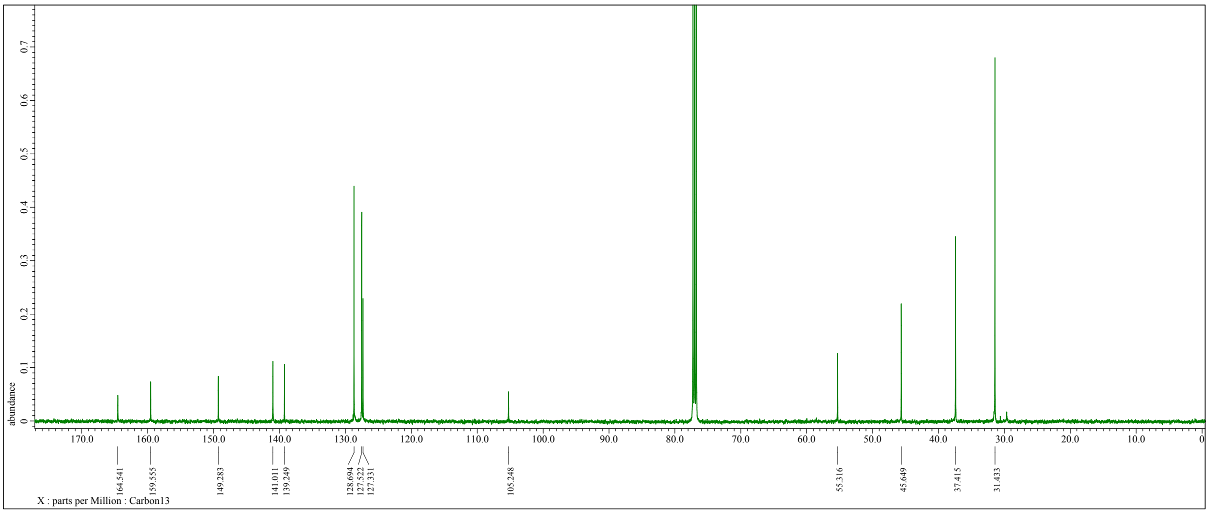

N-Benzyl-7-(*tert*-butyl)-2-methoxy-7H-purin-6-amine (**16**)

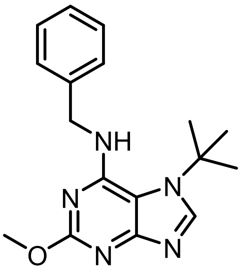

^{1}H NMR (400 MHz, CHLOROFORM-*d*) δ 7.98 (s, 1H), 7.38-7.28 (m, 5H), 5.25 (t, *J* = 4.9 Hz, 1H), 4.84 (d, *J* = 4.9 Hz, 2H), 4.02 (s, 3H), 1.70 (s, 9H)

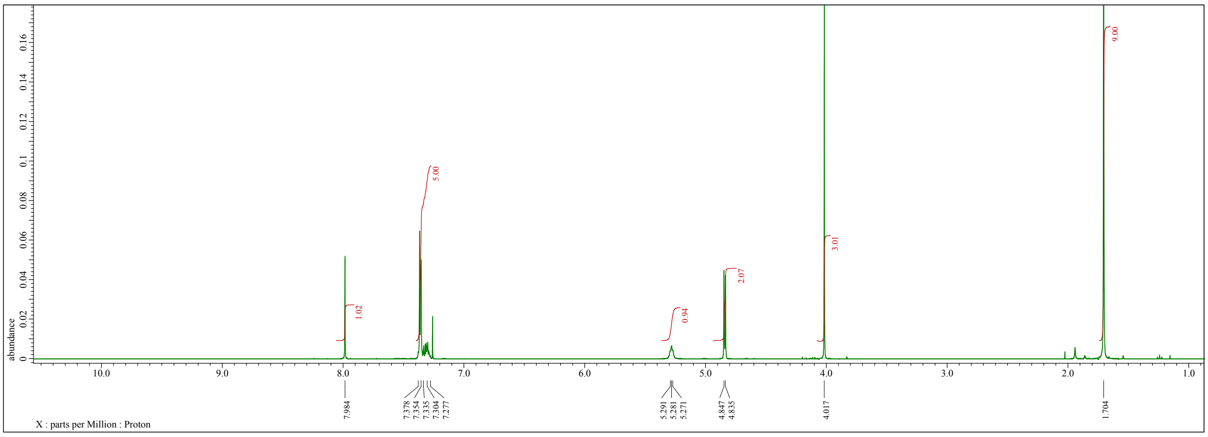

^{13}C{^{1}H} NMR (101 MHz, CHLOROFORM-*d*) δ 163.8, 162.0, 150.6, 141.8, 138.1, 128.9, 127.7, 127.7, 108.1, 55.9, 54.5, 46.0, 31.5

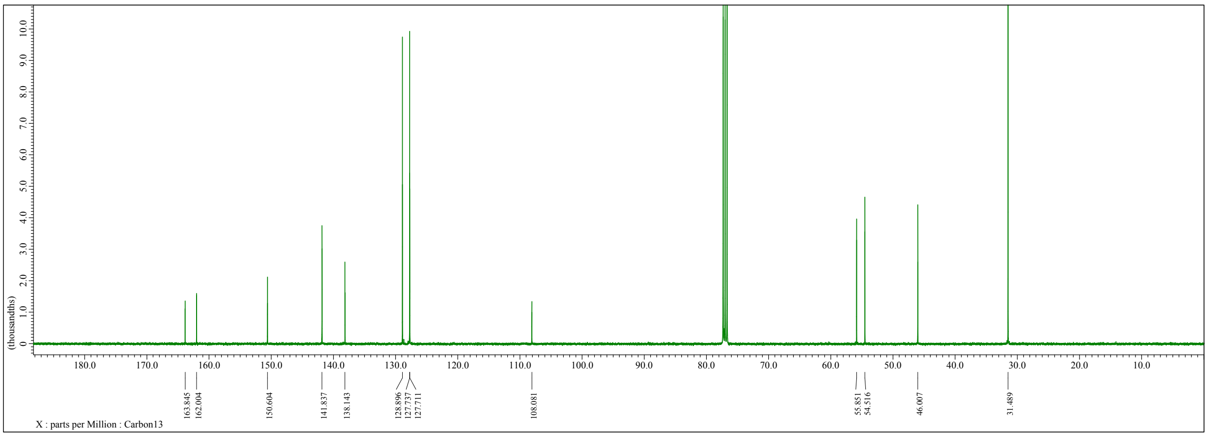

7-(*tert*-Butyl)-2-((3-hydroxypropyl)amino)-1,7-dihydro-6*H*-purin-6-one (**22**)

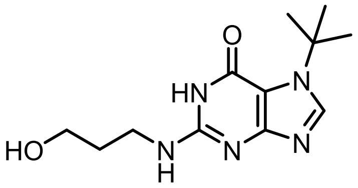

^{1}H NMR (400 MHz, CHLOROFORM-*d*) δ 11.80 (bs, 1H), 7.89 (bs, 1H), 7.82 (s, 1H), 3.78 (t, *J* = 5.6 Hz, 2H), 3.62 (q, *J* = 5.8 Hz, 2H), 1.89-1.83 (m, 2H), 1.74 (s, 9H)

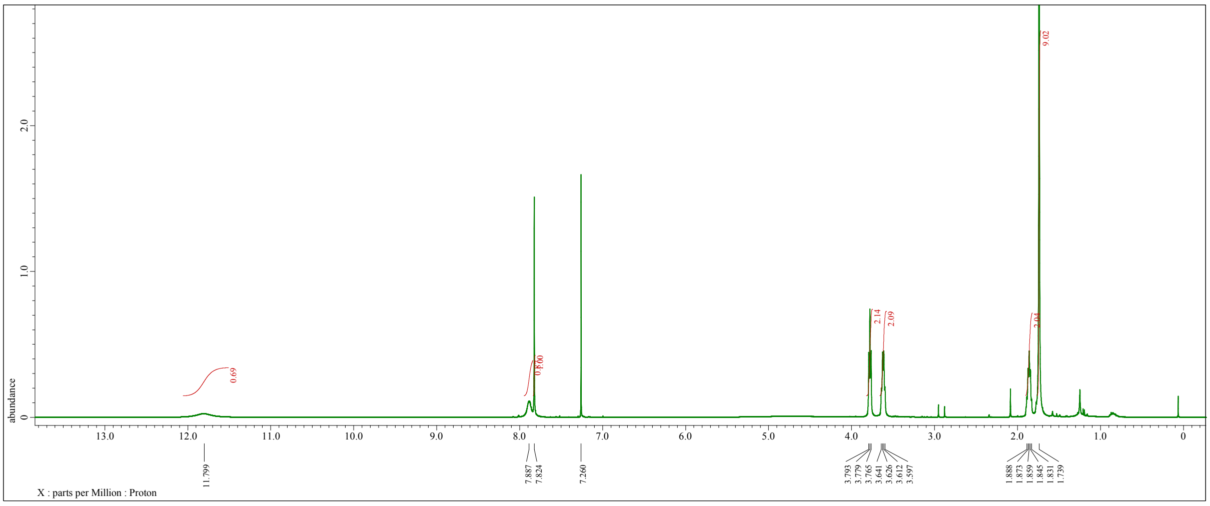

^{13}C{^{1}H} NMR (101 MHz, CHLOROFORM-*d*) δ 161.8, 155.7, 153.7, 140.1, 108.5, 58.6, 57.7, 37.6, 32.4, 29.7

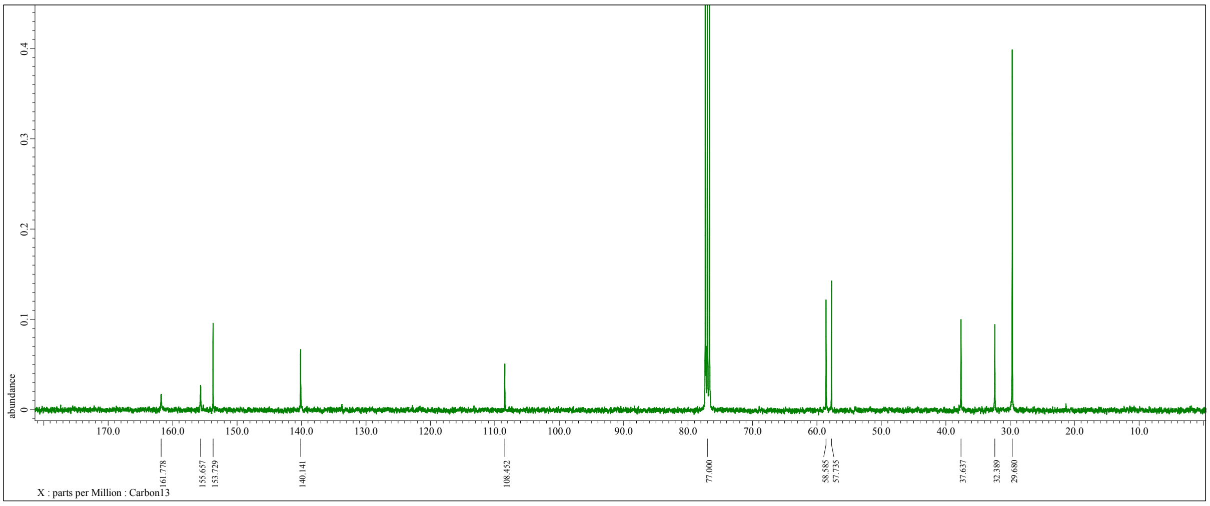

NOESY NMR spectrum of 7-(*tert*-butyl)-2-chloro-6-methoxy-*7H*-purine (**4**)

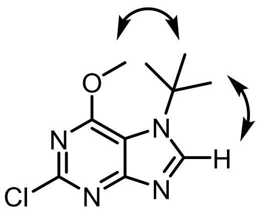

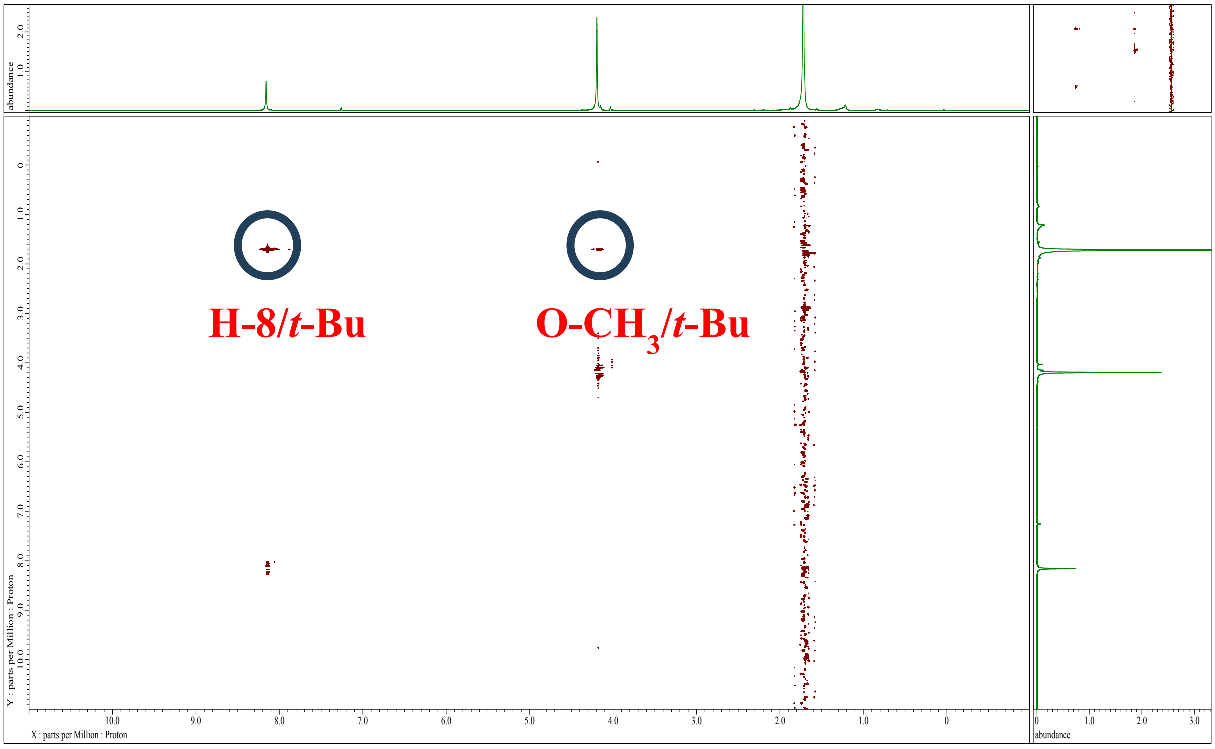

Supplement: Supplementary file 1 [file ao5c10284_si_001.pdf]
